# Discovery of RNA‐Targeting Small Molecules: Challenges and Future Directions

**DOI:** 10.1002/mco2.70342

**Published:** 2025-08-24

**Authors:** Zhengguo Cai, Hongli Ma, Fengcan Ye, Dingwei Lei, Zhenfeng Deng, Yongge Li, Ruichu Gu, Han Wen

**Affiliations:** ^1^ DP Technology Beijing China; ^2^ School of Mathematics Harbin Institute of Technology Harbin China; ^3^ Center for Quantitative Biology Academy for Advanced Interdisciplinary Studies Peking University Beijing China; ^4^ School of Pharmaceutical Sciences Peking University Beijing China; ^5^ Program in Molecular Medicine The Hospital for Sick Children Research Institute Toronto Ontario Canada; ^6^ Department of Molecular Genetics University of Toronto Toronto Ontario Canada; ^7^ State Key Laboratory of Protein and Plant Gene Research School of Life Sciences Peking University Beijing China; ^8^ Beijing Advanced Center of RNA Biology (BEACON) Peking University Beijing China; ^9^ AI for Science Institute Beijing China; ^10^ Institute for Advanced Algorithms Research Shanghai China

**Keywords:** bioactive small molecules, computer‐aided design, machine learning, RNA:protein interactions, RNA‐degrader, RNA‐targeting

## Abstract

RNA‐targeting small molecules represent a transformative frontier in drug discovery, offering novel therapeutic avenues for diseases traditionally deemed undruggable. This review explores the latest advancements in the development of RNA‐binding small molecules, focusing on the current obstacles and promising avenues for future research. We highlight innovations in RNA structure determination, including X‐ray crystallography, nuclear magnetic resonance spectroscopy, and cryo‐electron microscopy, which provide the foundation for rational drug design. The role of computational approaches, such as deep learning and molecular docking, is emphasized for enhancing RNA structure prediction and ligand screening efficiency. Additionally, we discuss the utility of focused libraries, DNA‐encoded libraries, and small‐molecule microarrays in identifying bioactive ligands, alongside the potential of fragment‐based drug discovery for exploring chemical space. Emerging strategies, such as RNA degraders and modulators of RNA–protein interactions, are reviewed for their therapeutic promise. Specifically, we underscore the pivotal role of artificial intelligence and machine learning in accelerating discovery and optimizing RNA‐targeted therapeutics. By synthesizing these advancements, this review aims to inspire further research and collaboration, unlocking the full potential of RNA‐targeting small molecules to revolutionize treatment paradigms for a wide range of diseases.

## Graphical abstract

This review explores challenges and future directions in discovering RNA‐targeting small molecules, from analyzing RNA structures and computational design to experimental workflows. It discusses mechanistic strategies like splicing modulation and RNA degradation, alongside advanced tools (deep learning, docking, and validation) to accelerate drug discovery. The discussion bridges structural insights, therapeutic targeting, and emerging technologies to advance RNA‐focused therapeutics.

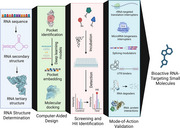

## 1 Introduction

The ENCODE project [1] has revealed that the majority of the human genome is transcribed into RNA, with only a limited proportion (∼1.5%) being protein coding. While the prevailing paradigm in drug discovery has focused on identifying and targeting disease‐relevant proteins, this approach is increasingly constrained by the finite number of protein targets [2] and their druggability [3], as evidenced by the escalating costs [4] associated with new drug development. However, advancements in RNA biology and structural characterization have positioned RNA targeting as an emerging field in drug discovery. Early milestones, such as the identification of antibiotics like aminoglycosides that bind ribosomal RNA [5] and metabolite analogs binding to regulatory riboswitches [6], demonstrated the potential of RNA‐targeting small molecules. Despite these advances, the field has faced significant challenges [7, 8], including RNA's structural flexibility, limited high‐resolution structural data, and the complexity of RNA–ligand interactions. Addressing these challenges requires a thorough understanding of both the biological pathway associated with the target RNA and the RNA–ligand interactions that can inform the design of selective, potent chemical probes to modulate the pathway.

Although RNA‐targeted drug discovery is an emerging field, the toolkit for characterizing and understanding RNA‐binding events remains underdeveloped and lags far behind protein‐focused methods. The effectiveness of RNA‐targeting is often debated, partly due to the lack of robust assays capable of detecting transient and seemingly weak binding interactions. Nevertheless, even currently United States Food and Drug Administration (US FDA)‐approved drugs have been found to interact pervasively with the human transcriptome, which may contribute to their pronounced side effects [9]. The fact is that current target engagement studies predominantly examine protein interactions, but largely overlook potential RNA‐mediated mechanisms. Therefore, fully harnessing the potential of RNA‐targeted therapeutics will require sustained commitment from both the scientific community and funding agencies to overcome these methodological and conceptual hurdles.

Notwithstanding the current conundrum, recent progress in RNA‐related biotechnologies has reinvigorated the field, enabling innovative RNA‐targeting therapies for diseases. Emerging strategies, such as DNA‐encoded libraries (DELs) [10] and fragment‐based screening methodologies [11], are effectively expanding the chemical space for RNA binders. Specifically, advances in machine learning [12], molecular dynamics (MD) and physics‐based modeling [13] are now accelerating the rational design of RNA‐targeted compounds, offering an efficient alternative to conventional approaches for RNA ligand design. After decades of research, splicing modulation has emerged as the most clinically validated strategy [14, 15], exemplified by the US FDA‐approved drug risdiplam [16] and various splicing‐based candidates in current clinical trials. Beyond splicing, novel approaches are diversifying the therapeutic toolkit. Modalities such as targeted RNA degraders [17] and small molecules modulating RNA–protein interactions (RPIs) [18] are now opening new frontiers. Despite this momentum, challenges persist, including achieving high target specificity and structural novelty, and translating in vitro findings into clinical applications smoothly. Computational approaches that integrated with experimental validation are increasingly critical to address these gaps, offering a path toward more precise and tunable RNA‐targeted therapeutics. This review aims to synthesize these challenges and recent developments, providing a comprehensive overview of the field's progress and future directions.

The overall manuscript is structured to systematically explore the various landscape of RNA‐targeting small molecules. We begin with an examination of RNA structure determination, covering experimental and computational methods that underpin rational drug design. Next, we delve into bioactive small molecules targeting RNA, introducing their mechanisms of action and the related clinical and preclinical studies. The review then discusses computer‐aided ligand design, emphasizing the role of bioinformatics, cheminformatics, and artificial intelligence in accelerating discovery. Subsequent sections explore screening methodologies for hit identification, followed by innovative approaches like RNA degradation and modulation of RPIs. Finally, we conclude with prospects for the field, underscoring the potential of RNA‐targeted therapies to revolutionize treatment paradigms. By presenting this cohesive framework, we aim to inspire further research and collaboration in this promising area of drug discovery.

The rationale for this review lies in the rapid evolution of RNA‐targeting technologies and the need for a consolidated resource to guide researchers. While comprehensive reviews [7, 19, 20, 21, 22] have mostly documented principles and experimental techniques for the discovery of small molecules targeting RNA, and recent discussions have highlighted the growing role of artificial intelligence and computational tools in the era of data‐driven science, integrative discussions bridging both experimental and computational approaches remain underrepresented in the literature. At this critical juncture as the field gradually transitions from fundamental discoveries to clinical applications, synergizing computational and experimental efforts is key to advancing the field, making a comprehensive synthesis of current knowledge both essential and timely. This review not only highlights key achievements but also identifies unresolved challenges, offering insights into future opportunities. By bridging gaps between structural biology, computational science, and therapeutic development, we aim to foster interdisciplinary advancements that will unlock the full potential of RNA‐targeting small molecules in medicine.

## 2 RNA Structure Determination

RNA structure serves as the foundation for understanding RNA function and for the rational design of RNA‐targeted small molecules. This primarily encompasses the determination of RNA secondary structures as well as the detailed high‐resolution characterization of tertiary structures. Nevertheless, the inherent structural flexibility and polycationic nature of RNA pose significant challenges to conventional biophysical methods for characterizing RNA tertiary structures. Consequently, the number of successfully elucidated RNA structures remains limited, with a preponderance of those solved being ribosomal RNAs, transfer RNAs, and riboswitches [23]. In contrast, computational prediction of RNA structures has recently emerged as a complementary approach that has garnered increasing attention. Machine learning algorithms, trained on established RNA structures, are now capable of predicting secondary and tertiary structures with remarkable accuracy. These algorithms can integrate diverse data sources, such as sequence information, chemical probing data, and evolutionary conservation, to construct reliable structural models. Unlike experimental techniques, which are frequently constrained by time and resource limitations, computational approaches can simulate a multitude of possible conformations within a relatively short time frame, thereby offering valuable insights into the conformational ensemble that an RNA molecule may adopt.

In this section, advanced biotechnologies and computational algorithms on RNA structure determination will be discussed. Secondary structure prediction methods have evolved from nearest neighbor models to probabilistic models and, more recently, deep learning approaches. Chemical probing becomes a robust tool to gain insights of RNA structures by sensing chemical reactivity of the nucleotide (nt). Techniques like mutational profiling (MaP) and detection of RNA folding ensembles using expectation–maximization (DREEM) have advanced our understanding of RNA dynamics and interactions by coupling with statistical models. For high‐resolution 3D structure determination, X‐ray crystallography remains the gold standard, despite challenges in crystal formation. Various engineering techniques have been developed to enhance RNA crystal packing. Nuclear magnetic resonance (NMR) spectroscopy excels in studying smaller RNA molecules and their dynamics, employing strategies like selective labeling and “divide‐and‐conquer” approaches to overcome size limitations. 3D structure prediction methods, including traditional simulation‐based and template‐based approaches, as well as emerging deep learning techniques, are continuously improving. However, challenges remain due to RNA's conformational flexibility and the limited availability of high‐quality experimental data for model training and validation. Integrative approaches combining multiple experimental and computational methods show promise in overcoming individual limitations and providing comprehensive structural insights into complex RNA molecules [24]. As more RNA structures are solved and AI techniques advance, the field of RNA structure prediction and analysis is poised for significant progress, potentially accelerating discoveries in RNA biology and drug development.

### 2.1 Secondary Structure Prediction

RNA folding is characterized by a hierarchical organization of structural elements, wherein local secondary structures form initially, followed by the progressive assembly of higher‐order tertiary interactions [25]. The formation of secondary structures precedes and occurs at a significantly faster rate than the establishment of tertiary conformations [26]. The secondary structures of RNA are stable and play an important role in the functionality of many noncoding RNAs (ncRNAs) [27]. Importantly, the secondary structure alone is often sufficient to inform functional predictions and facilitate various practical applications, underscoring its importance in the context of RNA biology [28].

**FIGURE 1:**
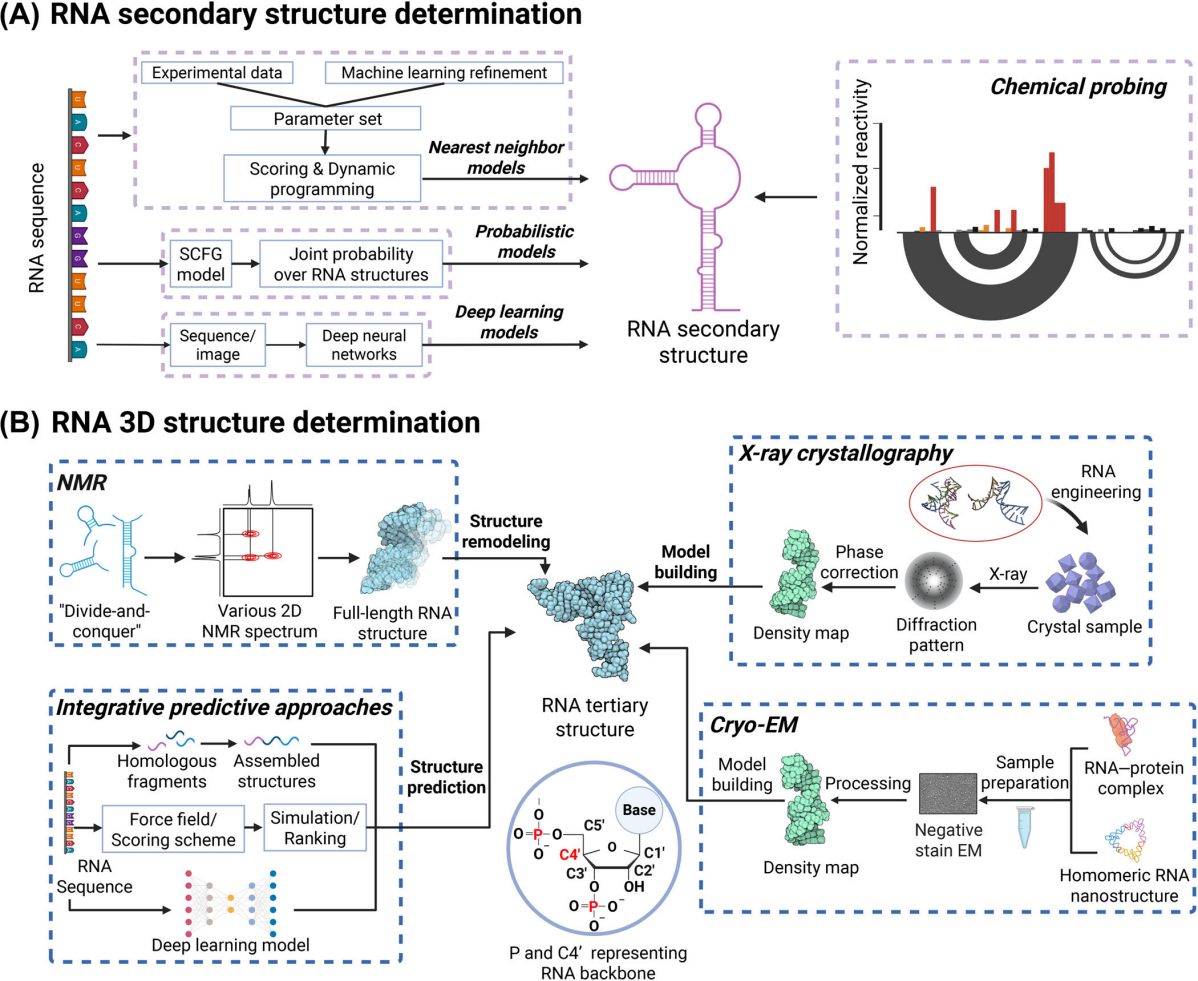
RNA structure forms foundation of RNA biology and RNA‐targeted therapeutics. (A) Most of computational approaches for RNA secondary structure predictions can be divided into three categories: nearest neighbor models, probabilistic models, and deep learning models. Chemical probing is the most prevalent experimental method to determine RNA secondary structures and shed light on the tertiary structure by probing interactions between distal nts. (B) Various RNA tertiary structure determination methods ranging from experimental approaches (e.g., X‐ray crystallography, NMR, and cryo‐electron microscopy (cryo‐EM) to computational approaches (e.g., traditional template and simulation‐based methods, as well as deep learning models that reconstruct the RNA structure directly or optimize it after predicting the scoring function), have contributed to the resolution of multiple RNA structures to date. Created with BioRender.com.

Since the 1970s, a diverse array of computational methods has been developed to predict the secondary structure of RNA molecules, including nearest neighbor models, probabilistic models, and deep learning models (Figure 1A).

#### 2.1.1 Nearest Neighbor Models

The nearest neighbor models can be used for the calculation of free energy changes induced by the secondary structure changes of RNA molecules. Using dynamic programming algorithm, the nearest neighbor model can be employed to find the minimum free energy structure, which is often considered as the native RNA structure.

The thermodynamic parameter used in the nearest neighbor model can be estimated from multiple experiments, such as optical melting studies or others [29]. The Nearest Neighbor Database (NNDB), developed by the Tuner group, is a web‐based resource for disseminating thermodynamic parameter sets for predicting secondary structures. NNDB covers parameters for predicting both RNA folding free energy and enthalpy changes [30]. Meanwhile, machine learning methods can also be introduced to refine the estimation of thermodynamic parameters [31, 32, 33, 34].

Based on the thermodynamic parameter estimated from experimental data and refined by machine learning methods, several nearest neighbor models were developed. RNAstructure leverages thermodynamic models and the nearest neighbor parameter set from the Tuner group for RNA secondary structure prediction [35]. RNA2Dfold in ViennaRNA package includes an implementation of partition function for computing base‐pair probabilities and has showed good performance on the prediction of circular RNA folding [36, 37]. The LinearFold algorithm employs an innovative dynamic programming approach to RNA secondary structure prediction, incorporating heuristics that facilitate linear time and space complexity (O(n)) [38]. While this method yields high accuracy in general, it is important to acknowledge that the results are approximate rather than exact. For nearest neighbor models, the dynamic programming algorithm exhibits a time complexity of O(n^3^), which becomes computationally prohibitive for sequences exceeding 1000 nts in length. Moreover, predicting the special base pairs including noncanonical base pairs, base triples and pseudoknots in RNA structures is still a difficult task for nearest neighbor methods.

#### 2.1.2 Probabilistic Models

Probabilistic models usually apply stochastic context‐free grammars (SCFGs) to predict RNA secondary structures. SCFGs specify formal grammar rules and include a joint probability distribution over possible RNA structures for a given sequence [39]. Followed by this strategy, many algorithms have been devised. For instance, Pfold improved the SCFG model performance by adding the evolutionary information to the context [40]. Contrafold proposed a nonparametric Bayesian extension of SCFGs with the hierarchical Dirichlet process to find an optimal RNA grammar from the training dataset [41]. Probabilistic models based on SCFG have inherent limitations in secondary structure prediction due to the fact that SCFG cannot describe all RNA structures, such as the structure containing special base pairs.

#### 2.1.3 Deep Learning Models

Deep learning has emerged as a powerful tool for the RNA secondary structure prediction in recent years. A plethora of efficacious deep learning models and algorithms have been developed to address this fundamental challenge in RNA biology, significantly advancing the predictive accuracy to unprecedented levels. SPOT‐RNA uses transfer learning strategy and an ensemble of deep hybrid network containing Residual Networks (ResNets) and 2D Bidirectional Long Short‐Term Memory networks (2D‐BLSTMs) for the structure prediction task [42]. By employing evolution‐derived sequence profiles and mutational coupling as inputs, SPOT‐RNA2 outperformed SPOT‐RNA for all types of base pairs in the task of RNA secondary structure prediction [43]. UFold proposes a novel image‐like representation of RNA sequences, which can be efficiently processed by fully convolutional networks [44]. 2dRNA is the representative of a type of length‐dependent model, which could be obtained by training a fundamental length‐independent model across various RNA length intervals through transfer learning [45]. GCNfold utilizes a three‐layer graph convolutional network (GCN) to mine the structural information of RNA motifs, encompassing stems, hairpins, and internal loops. The structural data of these RNA motifs are encoded via transformer encoders, while long‐range pairwise interactions are captured through the implementation of a UNet framework. Despite its compact design with a small set of parameters, GCNfold achieves rapid inference and maintains high accuracy, surpassing 80% among all models [46]. RNADiffFold considers the prediction of contact maps as a pixel‐level segmentation task and trains the denoise model to iteratively refine contact maps from noise. Experimental results on both within‐ and cross‐family datasets demonstrate RNADiffFold's competitive performance compared with current state‐of‐the‐art methods. Moreover, RNADiffFold moderately captures dynamic structural features of RNA, as validated on a multiconformational dataset [47].

### 2.2 Chemical Probing

Chemical probing techniques have significantly advanced our understanding of RNA structure and function, thereby playing a pivotal role in the broader landscape of RNA research (Figure 1A). These techniques, which primarily include dimethyl sulfate (DMS)‐based probing and selective 2′‐hydroxyl acylation analyzed by primer extension (SHAPE), have become cornerstones in the field, representing some of the most commonly utilized methods for probing the reactivity of functional groups within RNA structures. The underlying principle involves the introduction of adducts through chemical modifications to the RNA molecule, which are subsequently read out via reverse transcription experiments. Conventionally, the modified nt will hamper the reverse transcriptase to proceed to the next nt, leading to the truncated cDNA product.  To enhance the robustness and reproducibility of the sequencing readout, MaP strategies for both SHAPE and DMS probing were developed in separate investigations [48, 49]. By using specific reverse transcriptase or optimizing the reverse transcription condition, the termination of the reverse transcription could be resumed by incorporating an incorrect DNA base, leading to the mutational cDNA sequence, whose sequencing profiles will be used to determine the RNA structure.

Early SHAPE reagents, such as *N*‐methylisatoic anhydride, 1‐methyl‐6‐nitroisatoic anhydride, and benzoyl cyanide (BzCN), have certain limitations, particularly in the context of in‐cell probing. In contrast, DMS has demonstrated robust chemical reactivity in living systems, highlighting its unique advantages. This disparity in the applicability of different reagents spurred the development of optimized SHAPE reagents, including 5‐nitroisatoic anhydride [50] and acyl imidazoles such as 2‐methylnicotinic acid imidazolide [51], which have expanded the toolkit available for in‐cell probing. Despite these advancements, traditional chemical probing techniques have a notable drawback: they typically lead to the determination of an averaged RNA structure, failing to capture the inherent heterogeneity in RNA structure. To address this limitation, Rouskin et al. [52] developed an innovative algorithm termed “DREEM”. By applying DREEM to the entire HIV‐1 genome, they not only validated the protocol by confirming the existence of in vitro characterized alternative conformations of the HIV‐1 Rev responsive elements in living cells but also revealed the diverse conformations of RNA elements at critical splicing sites within the HIV‐1 genome. This discovery elucidates how such conformational diversity affects the ratio of transcript isoforms, thereby influencing RNA function at the genomic level.

Future directions in this area must account for the complexity uncovered by chemical probing techniques. There is a pressing need to develop more sophisticated methods that can not only probe RNA structures in vivo with greater precision but also accommodate the structural heterogeneity observed. This could involve the development of novel chemical probes that are more sensitive and specific, capable of capturing transient and low‐abundance RNA conformations.

### 2.3 X‐Ray Crystallography

X‐ray crystallography remains the preeminent methodology for elucidating the atomic‐level three‐dimensional structure of RNA in contemporary structural biology. Since the initial RNA structure determination [53] in the 1970s, approximately 1,26 RNA‐only structures and 2717 RNA–protein structures have been deposited in the Protein Data Bank (PDB) using this technique, as of 2025 January. Out of 862 RNA‐small molecule complex structures summarized in Harnessing RIBOnucleic acid—Small molecule Structures (HARIBOSS), 650 records were originated from X‐ray crystallography [54]. Despite the superior resolution afforded by this method, the preparation of diffraction‐quality RNA crystals presents significant challenges in resolving most RNA structures. The inherent conformational heterogeneity and densely charged backbone of RNA molecules impede effective intermolecular contacts necessary for crystal formation. Consequently, a variety of techniques have been developed to engineer RNA packing in crystals as a prerequisite for structural determination via X‐ray crystallography (Figure 1) [55].

The most fundamental RNA interaction to be engineered for enhancing intermolecular interactions is through Watson‐Crick base‐paired duplexes, with the packing of two RNA helices by terminal base pairs being prevalent in numerous documented RNA crystal structures [56]. Another frequently employed engineering technique utilizes hairpin loops as instrumental building units [55]. The implementation of kissing loop complexes, a natural RNA tertiary interaction motif, to design sequence‐specific loop‐loop contacts is crucial in various naturally occurring biological processes, such as ColE1 plasmid replication [57] and viral genome dimerization [58, 59].

There are certain innate RNA structures being successfully resolved through X‐ray crystallography without the need for complex RNA engineering, likely due to their inherent structural stability and reduced heterogeneity. Notable among these RNA targets are various riboswitches, which are mRNA regions comprising a ligand‐binding aptamer domain that detects small molecules such as metabolites, and an expression platform that modulates gene expression via conformational changes upon ligand binding. A primary example is Ribocil, a novel antibiotic that modulates the bacterial flavin mononucleotide (FMN) riboswitch. Crystallographic studies have revealed that only one isomer ((S)‐Ribocil‐B) of racemic Ribocil mixtures binds to the RNA in a U‐shaped conformation, interacting with specific bases and demonstrating additional stacking interactions compared with FMN [60]. Subsequently, structure‐based drug design was employed to enhance the inhibitory activity of Ribocil‐B, resulting in the development of Ribocil‐C [61], which exhibited an eightfold increase in potency compared with the lead compound.

Another significant RNA element resolved by X‐ray crystallography is the RNA triple helix [62], exemplified by the metastasis‐associated lung adenocarcinoma transcript 1 (MALAT1). Le Grice and collaborators integrated small‐molecule microarray (SMM) screening, biophysical techniques, and computational methods to facilitate the identification of compounds that stabilize the MALAT1 element for nuclear expression (ENE) [63]. These compounds have demonstrated efficacy in modulating MALAT1 levels in cellular contexts and influencing downstream targets. However, elucidating the biological mechanisms underlying the poly(A) protection conferred by the triplex structure remains a significant challenge. The expanding knowledge of ENE structures and the discovery of novel chemical tools may pave the way for innovative drug discovery programs targeting RNA structures.

X‐ray crystallography is the leading method for determining atomic‐level 3D RNA structures, contributing the greatest number of RNA‐only, RNA–protein, and RNA‐small molecule complex structures deposited in databases, among other methods. Future research should focus on developing more advanced techniques for engineering RNA crystal packing, perhaps leveraging computational methods to predict optimal packing configurations. Meanwhile, combining X‐ray crystallography with other structural determination methods (e.g., cryo‐EM, NMR) in a more integrated manner could provide a more comprehensive understanding of RNA structures and their interactions, thereby overcoming the limitations of individual techniques and enhancing the design of RNA‐targeting therapeutics.

### 2.4 NMR

NMR spectroscopy serves as a versatile tool in structural biology, enabling the elucidation of atomic‐resolution structures of RNA under solution conditions that closely mimic native environments. While X‐ray crystallography excels in deciphering large and complex RNA structures, NMR spectroscopy demonstrates particular efficacy in investigating the structure and dynamics of relatively shorter RNA molecules. However, several challenges exist in RNA structure determination via NMR, stemming from factors such as low proton density, limited chemical diversity of monomeric units leading to significant signal overlap, and shorter transverse relaxation times of RNA resonances due to ^13^C‐attached protons (a common isotopic labeling) and more anisotropic shapes compared with globular proteins [64]. Consequently, the average size of NMR‐derived RNA structures is 29 nts, with the largest structure determined by NMR being 155 nts, and fewer than ten structures exceeding 100 nts deposited in the PDB [65].

The large size of the RNA, could pose challenges such as short T2 relaxation times and spectral overlap, which would have hindered complete assignments and the acquisition of experimental constraints [66]. To address issues of signal overlap and simplify NMR spectroscopy of large RNAs, various approaches have been developed. The most prevalent strategy employs a “divide‐and‐conquer” methodology (Figure 1B) [67]. This approach is particularly effective for elongated structures such as stem‐loops, where interactions between different segments are often absent or limited. The absence of long‐range interactions can be confirmed by comparing NMR spectra of separate fragments or analyzing linewidth and relaxation rates [66]. In such cases, individual domains rotate independently, resulting in less efficient relaxation mechanisms and well‐resolved NMR signals, a phenomenon also observed in multidomain proteins. This strategy was effectively demonstrated by Gabriele et al. in their study of a 110‐nt RNA thermometer from Neisseria meningitidis, with a functional core of 70 nts [68]. The 70‐nt RNA core structure was divided into three overlapping sections, each corresponding to different structural elements of the RNA: the upper helix, lower stem, and an overlapping middle stem. By superimposing the base‐pairs of these segments, the complete RNA structure could be reconstructed.

To understand RNA's role in biological pathways and facilitate RNA‐based drug discovery, determining its dynamic ensemble at atomic resolution is crucial. NMR spectroscopy, combined with computational modeling, provides a rich source of ensemble‐averaged measurements. Al‐Hashimi et al. [69] developed a rapid and accurate workflow to determine atomistic RNA dynamic ensemble models using NMR data and computational modeling. Their pipeline generates an RNA conformation library using rosetta fragment assembly of RNA with full‐atom refinement (FARFAR) structure prediction, which can be refined using NMR residual dipolar coupling data. The accuracy of the generated ensemble models is validated through quantum‐mechanical calculations of NMR chemical shifts and cross‐validation analysis. Furthermore, the same group demonstrated that ensemble‐based virtual screening (VS) could significantly enrich hit compounds against HIV‐1 TAR, achieving comparable hit rates to scenarios where RNA holo structures were used for VS [70].

### 2.5 Cryo‐EM

Cryo‐EM has the ability to provide 3D structural information of biological molecules and assemblies by imaging noncrystalline specimens (single particles) [71]. In a typical cryo‐EM single particle analysis (SPA) workflow, the biological specimens are prepared using techniques such as negative staining and vitrification so it survives the vacuum of the electron microscope, then 2D images of particles are then acquired and processed to form final 3D density maps (Figure 1B).

The determination of protein‐free RNA cryo‐EM structures faces several challenges including lack of effective approaches for obtaining properly folded RNAs with stable tertiary structures, as well as the small size and intrinsic heterogeneity of RNA molecules [72]. New approaches have been developed to overcome these challenges. Qu et al. [73] utilize RNA binding protein LtrA to stabilize the RNA structure and resolve 704 nt of the 902‐nt LtrB using cryo‐EM SPA. Liu et al. [74] use a nanostructure assembly strategy called RNA oligomerization‐enabled cryo‐EM via installing kissing loops to obtain homomeric self‐assembled dimers and trimers of protein‐free RNAs for SPA.

Hybrid approaches combining cryo‐EM and other experimental or computational methods were also developed to facilitate RNA structural analysis. Zhang et al. [75] used cryo‐EM map to restrain NMR model refinement to derive atomic ensembles of a 30 kDa HIV‐1 dimer initiation site RNA (DIS). This synergistic methodology facilitated the identification of notable structural features, including a flipped‐out nt at both ends of the duplex. MD simulations of multiple time points on simulated cryo‐EM data of DIS suggested that the intrinsic RNA structural heterogeneity limits cryo‐EM SPA from achieving higher resolution for the DIS RNA [75]. Li et al. [76] resolved the three‐dimensional structure of the full‐length T‐box‐tRNA^Gly^ complex at a resolution of 4.9 Å. In the cryo‐EM structure, central tRNA^Gly^ is resolved to 4.1 Å but the outer T‐box is only resolved to 6 Å, posing great challenges for RNA modeling especially in the newly identified 3′‐discriminator regions with previously unknown structure. They solved this problem by refining the moderate resolution cryo‐EM RNA model using co‐crystal structure of tRNA^Gly^ in complex with 3′‐discriminator from another bacterial species at 2.7 Å resolution. This integrative approach, combining cryo‐EM with X‐ray crystallography data, enabled researchers to overcome resolution limitations and provided comprehensive structural insights into this intricate regulatory RNA complex.

### 2.6 3D Structure Prediction and Integrative Approaches

The prediction of three‐dimensional RNA structures is more challenging than that of proteins due to the conformational flexibility inherent in RNA molecules. The local 3D structure of RNA is described by 7 torsion angles of each residue instead of 3 in proteins. Moreover, experimental measurements of RNA tertiary structures exhibit a substantial deficit in comparison with the elucidation of protein three‐dimensional structures, reflecting a significant impediment to computational prediction methodologies due to data paucity, especially on the high‐quality RNA structures [77]. Notwithstanding these challenges, tremendous efforts have been invested in the development of computational methods for the RNA 3D structure prediction, which can be categorized into two main categories: traditional approaches and deep learning approaches.

Traditional approaches include simulation‐based approaches and template‐based approaches (Figure 1B). For the simulation‐based approach, one usually builds a coarse‐grained model and a corresponding force field to simulate the folding process as well as to obtain the thermodynamic properties. SimRNA uses five virtual atoms to represent a nt, with P and C4′ atoms representing the backbone, and three virtual atoms representing bases and simulates RNA folding with Monte Carlo (MC) and simulated annealing [78]. In contrast, HiRE‐RNA is a six‐ or seven‐bead high‐resolution coarse‐grained model that can capture more atomic details to fold the RNA structure with better accuracy [79]. BRiQ construct a knowledge‐based potential by performing quantum‐mechanical calculations to reweight base‐base statistical potentials [80]. This approach aims to minimize possible effects of indirect interactions and thus provides a robust improvement in refining near‐native RNA models generated by a wide variety of modeling techniques.

Template‐based approach builds the 3D structure of a target RNA by using the known 3D structure of homologous molecules as templates, or by assembling the structure of its fragments using 3D structures of similar‐sequence fragments from known structures. After assembling the structure fragments, further optimization and scoring are performed to refine the assembled structure. Fragment assembly of RNA with FARFAR uses three‐nt segments as blocks and uses the MC strategy to assemble short fragment templates under the guidance of knowledge‐based energy functions [81, 82, 83]. FARFAR2 improves its speed and accuracy by combining updated fragment libraries and helix modeling [84]. RNAComposer uses the nt cycle modulus as blocks and 3dRNA uses the nt cycle modulus with one more base pair from connected stems as blocks [85, 86]. While these methodologies have demonstrated efficacy in certain RNA instances, the limited RNA fragments, the dearth of atomic‐level structural information, coupled with the inaccuracy of scoring functions, present substantial obstacles for them to obtain accurate prediction of tertiary structures, especially for RNA molecules longer than 200 nts.

Deep learning approach either directly predicts the structure of the RNA molecule or predicts the structure by constructing the score function using deep learning and then optimize the structure based on the score function (Figure 1B). E2Rfold‐3D is a fully differentiable end‐to‐end learning model which allows informative representation of the sequence and secondary‐structure‐assisted self‐distillation [87]. Recently a deep‐learning‐based method termed atomic rotationally equivalent scorer (ARES) was reported. ARES takes a structural model as input, atom features are repeatedly updated based on the features of nearby atoms, resulting in a set of features encoding each atom's environment. Features averaged across all atoms are fed into additional neural network layers, outputting the predicted root‐mean‐square deviation (RMSD) of the input from the true structure. To perform structure prediction, ARES is used to rank candidate structural models (e.g., generated by FARFAR2), identifying models that ARES predicts to be most accurate. ARES outperforms previous methods while being trained with only 18 known RNA structures, indicating that it can learn effectively from small datasets, overcoming a major limitation of standard deep learning methods [88]. It marks a breakthrough in the use of deep‐learning‐based models for RNA tertiary structure prediction. trRosettaRNA builds the RNA structure by Rosetta energy minimization, with deep learning restraints from a transformer network (RNAformer) [89]. AlphaFold2 revolutionized protein structure prediction through unprecedented accuracy [90]. Building on this breakthrough, AlphaFold3 expands predictive capabilities to diverse macromolecular assemblies, encompassing RNA–ligand complexes and standalone RNA structures [91]. Comparative studies show that AlphaFold3's performance is comparable to other machine‐learning‐based methods in predicting RNA structure existing in RNA‐small molecule complexes albeit with some challenges in accurately modeling ligand binding sites [92]. When predicting the structure of large RNA molecules, the results returned by AlphaFold3 often contain severe steric clashes and occasional discontinuities in phosphodiester backbone connectivity. Thousands of predictions may be required to obtain plausible models for RNAs approaching 2000 nts in length [93]. Some later derivatives of AlphaFold3 such as Boltz‐1 [94] and Protenix [95] follow the general framework of AlphaFold3 but also introduce several refinements in the methodology. They achieve similar performance with AlphaFold3 and are open‐source, empowering researchers to drive further advancements in this field. Following Boltz‐1, Boltz‐2 is a new open‐source structural biology foundation model introducing controllability features including experimental method conditioning, distance constraints, and multichain template integration for structure prediction [96]. Boltz‐2 exhibits strong performance for both structure and affinity, providing a robust and extensible foundation for both academic and industrial research.

In summary, RNA structure determination faces significant challenges due to RNA's inherent flexibility, conformational heterogeneity, and limited high‐resolution experimental data. While traditional methods like X‐ray crystallography and NMR provide atomic‐level insights, they struggle with dynamic or large RNA molecules. Computational approaches, particularly deep learning, show promise but are hindered by scarce training data. Future advances necessitate combining experimental and computational approaches to study RNA dynamics while growing structural datasets to support the training of more predictive models.

## 3 Bioactive Small Molecules Targeting RNA

Bioactive small molecules targeting RNA represent a rapidly growing class of therapeutics, capable of modulating gene expression through diverse mechanisms, from interrupting ribosomal translation to altering pre‐mRNA splicing or microRNA (miRNA) biogenesis, and binding to the untranslated regions (UTRs) of the mRNA. These molecules exploit unique structural features of RNA, such as hairpins, bulges, and internal loops, to achieve selective binding and functional modulation. Recent advances have demonstrated their therapeutic potential across a broad spectrum of diseases, from infectious diseases and genetic disorders to cancers. With multiple candidates in clinical trials and preclinical development, RNA‐targeted small molecules are poised to re‐define treatment paradigms. Challenges remain in terms of compound selectivity, toxicity, and translating in vitro findings to clinical applications, but the integration of structural biology, computational design, and robust validation of mechanisms in complex biological systems continues to drive breakthroughs. This section explores key advances in bioactive small molecules targeting ribosomal RNA, pre‐mRNA splicing, miRNA biogenesis, and mRNA UTRs, highlighting their therapeutic potential, mechanistic insights, and clinical progresses.

### 3.1 rRNA‐Targeted Translation Interrupters

**FIGURE 2:**
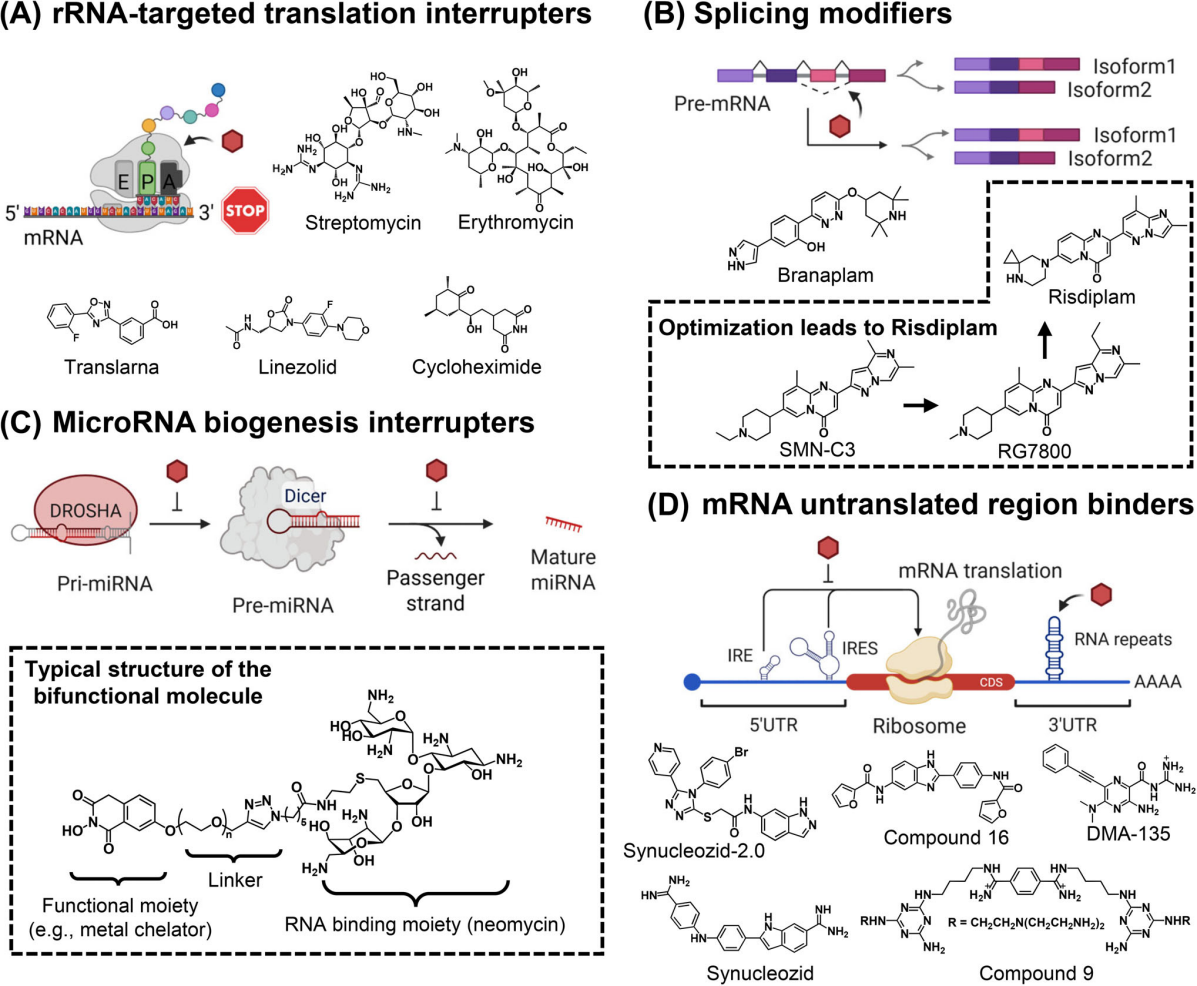
Different mode‐of‐actions for bioactive small molecules targeting RNA. Representative small molecules with their chemical structures were provided for (A) rRNA‐targeted translation interrupters. (B) Splicing modulators. (C) MiRNA biogenesis interrupters. (D) mRNA untranslated region binders. Created with BioRender.com.

As the essential component of ribosomes, rRNAs account for >80% of cellular RNAs and are folded into functional pockets and clefts accessible for small molecules [22]. Its abundance and accessibility lay solid foundation for rRNA‐targeted translation‐interrupting therapies. One well‐established application amongst is antibiotics. As one of the most conserved “hallmark” genes, rRNA bears substantial differences between prokaryotes and eukaryotes, ensuring the selectivity. Aminoglycoside‐based antibiotics, for example, streptomycin, which mediate translation misreading by direct binding of bacterial ribosomes 16S rRNA's aminoacyl‐site (A‐site) on 30S subunit, exert their specificity by leveraging a single nt difference (A1408G) between prokaryotic and eukaryotic ribosomes (Figure 2A) [97].

Another family of antibiotics, tetracyclines, bind to the same region as aminoglycosides and inhibit protein synthesis by hindering tRNA binding [98]. However, their selectivity are mainly attributed to difference in cytoplastic ion conditions (especially Mg^2+^ concentration), which grant them versatility against not only bacteria but also protozoa and helminths [98]. Other functional sites on rRNA are also identified as druggable pockets. For instance, macrolides (e.g., erythromycin), bind to the nascent peptides exit tunnel of the 50S subunit and interrupt translation elongation, and its selectivity is attributed to the specific conformation of the binding site within the peptidyl transferase center on the 50S subunit and the unique interaction with rRNA and ribosomal proteins in this region [99]. While all aforementioned therapies are derived from natural products, synthetic antibiotics exist to act on ribosomes. Oxazolidinones such as linezolid are first known as monoamine oxidase inhibitors in 1950s, identified as antibacterial agents back in 1970s, and not known to bind to domain V of the 23S rRNA of the 50S ribosomal subunit until late 1990s (Figure 2A) [100].

Moreover, ribosome‐targeting entities bear more potential than just pathogen inhibition. Eukaryote‐specific antibiotics like cycloheximide are shown to bear anticancer potential, as the high protein synthesis rate of cancer cells makes them extra sensitive to ribosomal inhibition [101]. Entities interfere with the synthesis of specific protein are also demonstrated to treat genetic disorder. Inspired by aminoglycosides’ readthrough‐promotion effects on premature nonsense codons in mammalian cells and their potential to increase the production of functional proteins in animal models of nonsense mutation diseases, Translarna, a selective nonsense suppressor with UGA/+1C preference, was found in high‐throughput screening (HTS) and shown efficacy to treat Duchenne muscular dystrophy in the animal model (Figure 2A) [102]. However, after its approval in Europe, clinical evidence only indicates marginal benefits for patients and its authorization is facing a nonrenewal [103]. Its application for US marketing is under intense scrutiny. The initial filing to the US FDA in 2016 received a complete response letter. Now, with the resubmission, the outcome remains uncertain as the US FDA reviews new data, which have potential implications for PTC Therapeutics [104].

### 3.2 Splicing Modifiers

Pre‐mRNA alternative splicing is a prevalent regulatory event that occurs in nearly 95% of human multiexon genes [105]. It is a crucial process in mammalian gene expression where specific exons in pre‐mRNA are selectively included or excluded, generating multiple protein isoforms from a single pre‐mRNA sequence. This process is of great physiological significance as it contributes to protein diversity. It can also lead to reduced translation of mRNAs through the introduction of premature stop codons via poison exons (PE) [106]. Alternative splicing is significantly involved in the development of numerous diseases. It causes genetic disorders like Fukuyama muscular dystrophy through SINE–VNTR–Alu retrotransposons‐mediated abnormal splicing [107]. In neurodegenerative diseases such as myotonic dystrophy and amyotrophic lateral sclerosis (ALS), it contributes to the disease process, by mis‐regulating trans‐acting factors [108, 109]. Moreover, in the context of many cancers, alternative splicing promotes tumorigenesis by generating oncogenic splicing isoforms that can enhance cell survival, proliferation, and metastasis [110].

To date, US FDA has approved only one small‐molecule drug targeting nonribosomal RNA, which modifies alternative splicing of the survival of motor neuron 2 (SMN2) for the treatment of spinal muscular atrophy (SMA) [16]. SMN2 is the paralogue of SMN1, whose deletion or mutation is the cause of SMA. Both genes produce the SMN protein, which has diverse functions, including the maintenance of normal motor functions. These two genes differ by a single silent C840T transition [111]. However, this transition results in alternative splicing, causing the exclusion of exon 7 from most SMN2 transcripts, which generates unstable SMN proteins. Given the fact that modest changes in SMN protein levels can have an impact on disease severity [112], modifying SMN2 splicing is considered a potential therapeutic approach for alleviating SMA.

SMN‐C3 was the first SMN2 splicing modifier identified by chemical screening and optimization (Figure 2B) [113]. It was further optimized into RG7800 [114], which demonstrated remarkable efficacy in preclinical SMA mouse models, increasing SMN2 mRNA and protein levels in both brain and muscle tissues [115]. Nevertheless, due to chronic retinal toxicity observed in animal experiments, the clinical trials of RG7800 were terminated [116]. Additional chemical modifications led to the successful development of risdiplam, which was approved by US FDA in 2020 [16] and became the first orally available treatment for SMA. Another compound named branaplam (Figure 2B) was developed under the same mode‐of‐action by Novartis around the same time. Both risdiplam and branaplam targets 5′‐ss of exon 7 in SMN2 pre‐mRNA, but probably with different binding sites and sequence preferences [117]. Ishigami and coworkers [118] utilized massively parallel splicing assays and RNA‐seq experiments to build quantitative models for explaining binding modes of risdiplam and branaplam. The results suggested that the binding specificity of risdiplam originated from a single IUPAC recognition motif (ANGA/GUHDNN). This simple and distinct recognition pattern allows it to more specifically target certain 5′ss sequences. In contrast, branaplam recognizes 5′‐ss sequences via two distinct interaction modes via two distinct IUPAC motifs (the risdiplam‐like IUPAC motif and the hyper‐activation IUPAC motif: NAGA/GUNNNN) [118]. This more dynamic recognition mechanism may lead to a broader range of target sequences, resulting in lower selectivity for a particular exon like SMN2 exon 7. A plausible explanation for the two‐interaction‐mode mechanism is that branaplam bears multiple tautomeric isoforms and high rotational degree of freedom, which allows it to adopt multiple bind conformations and orientations. In fact, both compounds entered clinical trials in 2015, but branaplam was halted in 2016 due to the adverse events observed in animal studies including the damage to nerves, the spinal cord, testes, and kidney blood vessels [119]. In mid‐2021, Novartis discontinued the further development of branaplam for SMA due to the highly competitive landscape within this therapeutic indication. Despite this setback in the SMA context, branaplam has demonstrated promise in reducing huntingtin mRNA levels, the mutated protein in Huntington's disease (HD), earning the US FDA Orphan Drug Designation for HD, with a phase IIb trial started in 2021 but soon terminated in 2022, due to the observed peripheral neuropathy and other side effects [120].

### 3.3 MiRNA Biogenesis Interrupters

miRNA are small noncoding RNAs about 22‐nt long, playing crucial roles in RNA silencing by targeting most protein‐coding transcripts. The binding site of miRNA is usually located at the 3′‐UTR while other interacting regions were also reported, including 5′‐UTR [121], coding sequence [122] and promoter regions [123]. miRNAs interact with target mRNAs through partial complementary base pairing, predominantly via nucleotides 2–8 of their sequence, also known as the seed region [124]. Over 60% of human mRNAs possess at least one conserved miRNA binding site [125]. Considering the potential nonconserved binding sites, most of protein‐coding genes are controlled by the miRNAs at translational level. Unsurprisingly, biogenesis of miRNA is rigorously regulated in cells, and dysregulation of miRNA is usually associated with human diseases, including cancers, psychiatric and neurological diseases, cardiovascular disease, and autoimmune disease [126].

The biogenesis of miRNAs is a multistep process (Figure 2C). It starts with transcription, which is mainly carried out by RNA polymerase II, leading to the primary miRNA (pri‐miRNA) harboring an internal hairpin‐loop structure. A typical pri‐miRNA consists of a stem of 33–35 bp, a terminal loop and single‐stranded RNA segments at both the 5′ and 3′ ends, which is recognized and processed within the nucleus by a tandem of RNA‐binding protein (RBP) DGCR8 and the RNAse III nuclease Drosha [127]. The DGCR8/Drosha complex trims the stem–loop structure of the pri‐miRNA, releasing a small hairpin‐shaped RNA of approximately 65 nts long, namely the precursor miRNA (pre‐miRNA). Pre‐miRNA is subsequently exported to the cytoplasm by exportin 5, where it is cleaved by another endonuclease Dicer to generate a double‐stranded miRNA duplex [128]. While the guide strand is loaded onto an Argonaute protein to form the RNA‐induced silencing complex (RISC), the passenger strand is ejected from the Argonaute protein and degraded. Strand selection in RISC loading is determined by the thermodynamic stability of the duplex ends and the 5′ nt of each strand [129]. During the miRNA biogenesis, the intermediate pri‐miRNA and pre‐miRNA forms robust secondary structures that play critical roles in their enzymatic processing. These RNA secondary motifs could form distinct pocket conformations in three‐dimensional space, facilitating selective recognition by small molecules. Functional small‐molecule binders can effectively intervene miRNA biogenesis, by specifically binding to the enzymatic cleavage sites, leading to the regulation of downstream biological pathways.

This concept of mode‐of‐action has been substantiated by a plethora of experiments, leading to the discovery of a handful of bioactive small‐molecule ligands that target miRNA biogenesis [130]. For instance, Velagapudi et al. [131] introduced a lead identification strategy called Inforna, to identify small molecules that inhibit miRNA biogenesis. Inforna database documented various RNA motif‐small molecule interactions as well as their fitness. The authors parsed sequences and secondary structures of all human hairpin precursor miRNAs through Inforna, leading to the identification of lead small molecules for 22 different disease‐associated miRNA precursors, based on the two criteria: the targetable motif had to be in a Drosha or Dicer processing site, and the miRNA had to be causative of disease. Out of the 22 miRNA precursors, pre‐miR‐96 was selected for the validation with compound 1, which formed the most avid interaction pair predicted by Informa. It was revealed that compound 1 selectively downregulated the expression level of mature miR‐96 and upregulated the related protein target (FOXO1), inducing cancer cell apoptosis. Moreover, the selectivity of compound 1 was comparable to the oligonucleotide‐based modality, demonstrating the robustness of Inforna in predicting small molecules capable of targeting RNAs, surpassing traditional methods like screening and chemical similarity searching [131]. In addition to the single‐site binding, one could utilize the structural motif near the Drosha/Dicer site to design a dimeric compound that binds to both sites, leading to the enhanced affinity and selectivity [132]. In fact, this multimodal approach has been utilized in several studies (Figure 2C). In addition to the primary moiety with RNA‐binding capabilities, the secondary moiety can have diverse functions. These functional components include another binding moiety with moderate binding affinity [133], the RNase inhibitor moiety with metal chelating property to disrupt enzymatic reactions [134], and the artificial nucleobase engineered to specifically recognize additional base pairing in the pre‐miRNA duplex [135]. One of the most well‐known bifunctional chimeras in RNA targeting with small molecule is perhaps the RIBOnuclease TArgeting Chimeras (RiboTACs) developed by Disney group. In RiboTACs, a selective RNA binder is conjugated with a degrader moiety that can act as the direct RNA degrader or recruit the endoribonuclease in situ to degrade the target RNA. More discussions on RiboTACs will be provide in Section 6.

In conclusion, the exploration of bioactive small molecules targeting miRNA biogenesis hold great promise. These small molecules could offer a means to modulate miRNA‐mediated gene regulation and present potential therapeutic opportunities for a wide range of diseases. The achievement of promising proof‐of‐concept outcomes in the development of miRNA biogenesis interrupters has sparked the interest of pharmaceutical startups. Saverna Therapeutics, for instance, launched a program with the goal of developing small‐molecule drugs capable of modulating biogenesis of miR‐155, an upregulated ncRNA that has been observed in patients with systemic lupus erythematosus [136].

### 3.4 Small Molecules Targeting mRNA UTRs

A recent survey on RNA‐targeted bioactive small molecules has revealed that most of documented chemical probes bind to the 5′‐ or 3′‐UTR of cellular mRNAs or viral RNAs [137]. A plausible reason for UTRs being suitable targets by small molecules and attract great research interests is that UTRs are engaged in various posttranscriptional regulatory pathways, including RNA localization, stability and translation efficiency [138]. From the perspective of RNA structure, UTR typically harbors plentiful well‐structured RNA motifs. These motifs not only facilitate selective interactions between RNA and other macromolecules or metabolites but also provide binding sites for small molecules.

For instance, the internal ribosomal entry site (IRES) element, perhaps the best understood example of RNA structure and function in the translation control, is characterized with short linear motifs (e.g., poly(U) tracts) or structured elements (e.g., stem‐loops and pseudoknots) (Figure 2D). First identified in viral genome, IRES element directs initiation factors assembly and ribosome loading, through multiple RNA–RNA and RPIs. Various types of viruses take advantage of IRES‐dependent translation to directly hijack host translational machinery to produce essential proteins, which is likely a natural evolution result with such a limited genome capacity. In eukaryotes, while most of transcripts are modified with 5′‐cap and 3′‐poly (A) tail for canonical cap‐dependent translation, some transcripts do bear IRES elements for the complementary translation under stress conditions, such as starvation, endoplasmic reticulum (ER) stress, hypoxia, and viral infection. The first IRES element was found in eukaryotic mRNA that encodes immunoglobulin heavy chain‐binding protein [139], an essential component during viral infection. The interplay between IRES structure and its function makes it an attractive target for small‐molecule intervention by blocking essential IRES‐dependent translations. For instance, the 5′‐UTR of enterovirus 71 (EV71) contains six stem loops (SLs), and SLs II–VI promote cap‐independent translation via the IRES [140]. The SLII domain, in particular, interacts with multiple cellular proteins, and its sequence and structure are essential for EV71 replication, which was validated in mutation experiments [141]. Davila‐Calderon et al. [142] screened an RNA‐focused small‐molecule library against the EV71 SLII domain using a fluorescent indicator displacement (FID) assay, resulting a selective ligand (DMA‐135; Figure 2D) that binds to the bulge of SLII. The small‐molecule binding induced a conformational change of the RNA structure, and stabilized a ternary complex with AUF1 protein. In cell‐based studies, DMA‐135 attenuated IRES‐dependent translation and inhibited EV71 replication in a dose‐dependent manner with relatively low cellular toxicity. Dual‐luciferase assays showed that it specifically affected IRES‐driven translation, and plaque reduction assays confirmed its antiviral activity.

Another type of structural RNA regularly seen in 5′‐UTR is iron responsive element (IRE), which affects translation of a subset of mRNAs regulating iron homeostasis (Figure 2D) [143]. For example, the IRE in the 5′‐UTR of SNCA mRNA regulates α‐synuclein translation, a key protein in the pathogenesis of Parkinson's disease and other neurodegenerative disorders [144]. At low iron concentrations, iron regulatory protein binds to the IRE, repressing translation initiation by preventing ribosome scanning on the mRNA. Thus, targeting the IRE can potentially decrease α‐synuclein production by stabilizing IRE as a translation repressor for SNCA mRNA [145]. The IRE has a well‐defined three‐dimensional structure with elements like a 1×1–nt internal loop and a 1‐nt bulge with GC and GU closing base pairs. These structural features provide potential binding sites for small molecules. Additionally, the sequence of the IRE is highly conserved, with a low minor‐allele frequency, making it a stable target [146]. Zhang et al. [146] employed the Inforna as the sequence‐based design strategy, to identify an effective compound (Synucleozid; Figure 2D) to bind the A bulge near the base of the IRE hairpin. Binding assays, including 2‐AP‐labeled RNA and ASO‐Bind‐Map, were applied to confirm its specific binding to the A bulge in the IRE, both in vitro and in cells. Mechanistic studies using polysome profiling showed that Synucleozid specifically inhibits α‐synuclein translation by stabilizing the IRE, reducing ribosome loading onto SNCA mRNA and disrupting the preinitiation complex scanning. However, Synucleozid did not have ideal physiochemical properties for CNS penetration based on a druglikeness central nervous system multiparameter optimization (CNS‐MPO) algorithm [147]. A follow‐up study therefore screened a larger range of small‐molecule chemical space to afford a more potent compound (Synucleozid‐2.0; Figure 2D) with an enhanced CNS‐MPO score (3.5 when compared with 1.3 as for Synucleozid) [148]. Similar to the previous Synucleozid, Synucleozid‐2.0 bound to the same A bulge within the mRNA IRE, preventing ribosomal preinitiation complex formation as confirmed in the polysome profiling experiment. Compared with Synucleozid, Synucleozid‐2.0 has improved physicochemical properties for better blood–brain barrier penetrance, enhanced selectivity, more thoroughly validated binding site and mode of action, and greater efficacy in reducing α‐synuclein levels and conferring cytoprotection, with its RiboTAC derivative (Syn‐RiboTAC) showing even stronger effects [148].

The 3′‐UTR of mRNA contains cis‐regulatory elements that influence translation, stability, and localization, with mutations in this region linked to various diseases. Besides miRNA‐based regulation, many studies have suggested that disruptions in the 3′‐UTR, such as mutations in the termination codon, polyadenylation signal, or secondary structure, can dysregulate translation and contribute to certain disease [149]. One of the most studied RNA elements located in the 3′‐UTR might be the r(CUG) RNA repeating (r(CUG)^exp^; Figure 2D), which is the cause of the myotonic dystrophy type 1 (DM1) via a gain‐of‐function mechanism [150]. In healthy individuals, the 3′‐UTR of the dystrophia myotonica protein kinase (DMPK) mRNA normally contains 5–37 r(CUG) repeats, whereas DM1 patients may exhibit hundreds to thousands of these repeats. The excessively expanded r(CUG) repeats could sequester essential proteins such as muscleblind‐like 1 (MBNL1) and disrupt the pattern of pre‐mRNA alternative splicing, leading to the DM1 phenotype due to the splicing defect [151]. To address this, Rzuczek and coworkers screened an RNA‐focused small molecule library against r(CUG)^exp^ with a time‐resolved fluorescence resonance energy transfer (TR‐FRET) assay [152]. 28 initial hit compounds with 9% hit rate were identified via this assay, among which compound 1, 16, and 17 showed promise in a DM1 cellular model (Figure 2D). Further studies demonstrated that compound 16 and 17 directly bound r(CUG)^exp^ in cells, improving splicing defects without affecting DMPK mRNA levels, while compound 1 had a mixed mode of action [152]. The complexity of myotonic DM1 pathogenesis driven by toxic r(CUG)^exp^ in DMPK mRNA has spurred efforts to develop multitarget small molecules with distinct mechanisms of action operating simultaneously. Nguyen et al. [153] applied a multitarget strategy to tackle the complex pathobiology of DM1, using rational design based on the structural patterns of r(CUG)^exp^ and knowledge of cell‐permeable properties. The crafted small molecules to act in three ways: inhibit transcription by binding to CTG^exp^, prevent aberrant protein binding to CUG^exp^, and degrade CUG^exp^ as RNase mimics. Among these designed compounds, compound 9 (Figure 2D), which exhibited low toxicity, rescued the insulin receptor minigene splicing defect and regulated CUG^exp^ mRNA levels. In a DM1 Drosophila model, compound 9 improved neurodegenerative and locomotion phenotypes and reduced SV40 mRNA levels in larvae with disease‐length repeats [153].

The UTRs of mRNAs represent a rich yet underexplored frontier for small‐molecule drug discovery, offering structurally diverse motifs such as IRES elements, IRE hairpins, and repetitive expansions. Advances in rational design and HTS have yielded promising ligands that modulate translation, stability, or protein interactions by targeting these regions. Further discovery of biologically decisive RNA motifs within UTRs, particularly those governing disease‐specific regulatory switches, will be critical to expand the repertoire of druggable targets.

### 3.5 RNA‐Targeting Small Molecules in Clinical and Preclinical Development

Extensive research over decades has identified numerous RNA‐targeting small molecules with potent bioactivities, many of which are promising candidates for advanced preclinical or clinical evaluation. A notable example is Risdiplam, an approved therapeutic for SMA, which has spurred intensive investigation into splicing modulators. Beyond splicing modulation, small‐molecule inhibitors targeting mRNA have also been developed to modulate the expression of disease‐relevant transcripts, including those implicated in cancers and neurodegenerative diseases. To advance these findings toward therapeutic applications, several drug discovery pipelines have been established, focusing on this regulatory mechanism to achieve translational success. In contrast, ncRNAs face significant challenges in translational development despite their prevalence in the human genome and established roles in disease regulation. While compelling in vitro results have been reported, robust in vivo validation and clinical translation remain scarce, with few examples conclusively demonstrating therapeutic efficacy. This gap may stem from the inherently low expression levels of ncRNAs and their complex interaction networks, which hinder the progression of lead compounds through rigorous preclinical assessment. Nevertheless, the current status of RNA‐targeting small molecules in clinical and preclinical studies underscores their therapeutic potential. These advances encourage further exploration of innovative strategies, such as RNA degraders and covalent binders, to expand the scope of RNA‐targeting drug discovery in the realm of translational medicine.

In 2016, the US FDA granted approval to Nusinersen, marking the first disease‐modifying therapy for SMA [154]. This antisense oligonucleotide functions by modulating SMN2 pre‐mRNA splicing, thereby enhancing the production of full‐length SMN protein. However, its clinical application is limited by the necessity for intrathecal administration and high cost. Given these constraints, the development of orally bioavailable small‐molecule therapeutics has been pursued as a more patient‐friendly and cost‐effective alternative. In 2020, the US FDA‐approved risdiplam, an orally administered small‐molecule SMN2 splicing modifier, expanding the treatment options for SMA patients [16]. The novel mode‐of‐action by small molecules targeting pre‐mRNA alternative splicing events inspired scientists to search for other applicable indications, particularly HD.

HD is caused by an expanded CAG repeat in exon 1 of the Huntington (HTT) mRNA, leading to a toxic polyglutamine stretch in the HTT protein, resulting in HTT protein aggregation and a gain‐of‐function toxicity [155]. Thus, a promising therapeutic strategy involves reducing mutant HTT mRNA levels. Two small‐molecule splicing modulators, branaplam (Novartis) and PTC518 (PTC Therapeutics), were developed to alter HTT splicing by incorporating a PE, a sequence containing a premature termination codon that triggers nonsense‐mediated decay (NMD), degrading the mutant mRNA [156]. Both compounds showed efficacy in lowering toxic HTT protein level. However, the Phase 2b VIBRANT‐HD trial for branaplam was recently discontinued due to safety concerns, while PTC518 is nearing completion of its Phase 2 PIVOT‐HD trial (expected June 2024) [157]. It is noteworthy that US FDA has granted Fast Track designation to the PTC518, which is usually awarded to promising therapies for diseases of high unmet need [158].

The structure of PTC518 is undisclosed, but PTC Therapeutics has previously reported that risdiplam also targets HTT, albeit with lower potency [159]. Recent studies revealed that branaplam and risdiplam not only reduce Huntingtin protein via PE inclusion but also suppress CAG repeat expansion in HTT exon 1 by modulating PMS1, a known modifier of HD age‐at‐onset [156]. This suggests PTC518 may also have a dual‐target mechanism, simultaneously lowering HTT and PMS1 through alternative splicing modulation. Leading by PTC518, several other promising small molecules also successfully translated into clinical trials, including PTC‐607 (PTC Therapeutics) that currently in Phase 1 (ACTRN12622001534774) trial to assess its safety and tolerability in healthy participants, and SKY‐0515 (Skyhawk Therapeutics), is completing Phase 1 (NCT06873334) trial in this June, with Part A and B data of this clinical trial showed significant reduction of mutant HTT mRNA in healthy volunteers. The coming years will likely see a surge in clinical trials targeting mutant HTT, driven by companies such as Remix, Rgenta, and ReviR Therapeutics (see more updates on their official websites), which are now moving their most effective preclinical candidates into human testing.

Besides neurodegenerative diseases, the strategy of PE‐inclusion‐driven NMD could be applied to target other traditionally undruggable disease drivers, including oncoproteins overexpressed in cancers. Recently, Remix Therapeutics initiated two clinical trials to evaluate the safety and antitumor effects of its small‐molecule mRNA degrader (REM‐422) on the patients with recurrent or metastatic adenoid cystic carcinoma (ACC; NCT06118086) and acute myeloid leukemia/high‐risk myelodysplastic syndromes (AML/MDS; NCT06297941). REM‐422 potently reduces the level of c‐MYB (MYB) oncogenic transcription factor, by inducing the inclusion of a normally unused PE to the MYB mRNA [160]. In NOG mice engrafted with AML patient cells, daily oral REM‐422 (10 mg/kg) for 24 days eradicated human leukemia cells in bone marrow and peripheral blood, as confirmed by flow cytometry [160]. The same strategy has been applied to the development of RGT‐61159 [161], another oral inhibitor of MYB via PE inclusion, which is currently under Phase 1 clinical trial for the treatment of ACC (NCT06462183) and IND enabling for AML.

Small molecules can also be utilized to target RNA–protein quaternary structures to deliver potential therapeutic applications. The best example for this type of mode‐of‐action is probably the Zotatifin or eFT226. Zotatifin binds to the interface between eukaryotic translation initiation factor 4A (eIF4A) and mRNA with sequence preference for polypurine motifs at the 5′‐UTR, stabilizing the incompetent RNA/eIF4A complex [162]. Since eIF4A plays a critical role in unwinding mRNA secondary structures to facilitate ribosome scanning and translation initiation, this stabilization disrupts the 43S preinitiation complex's ability to locate the start codon (AUG), leading to selective translational suppression [163]. Given that many oncogenes depend on eIF4A‐mediated translation for their overexpression, inhibiting this process can effectively impair tumorigenesis and cancer cell survival. In a Phase 1/2 trial (NCT04092673), zotatifi demonstrated antitumor activity and a favorable safety profile (primarily grade 1/2 adverse events) when combined with fulvestrant or combined with fulvestrant and abemaciclib in heavily pretreated ER+ metastatic breast cancer patients, with partial responses observed in both cohorts [164]. Alternatively, small molecules can function as molecular glues to stabilize the interaction between eukaryotic ribosomes and eRF1, a critical translation termination factor responsible for stop codon recognition [165]. For instance, SRI‐41315 induces eRF1 degradation by binding to RNA–protein quaternary structures, thereby suppressing NMD through premature termination codon readthrough [166]. Although this compound has not yet entered clinical trials, it represents an alternative strategy for modulating disease phenotypes through RNA‐targeting small molecules.

The following table (Table 1) summarizes relevant preclinical and clinical studies on RNA‐targeting small molecules; although some studies might be missed unintentionally, we direct readers to other specialized reviews for further details [14, 21, 167].

**TABLE 1:** Small‐molecule drugs approved for clinical practice or under development that act on RNA targets.

Compounds	Molecular targets	Mechanisms of action	Clinical application	Current status
Risdiplam	*SMN2*	Modifies alternative splicing to increase SMN protein expression	SMA	Approved for medical use
Branaplam	*SMN2, HTT*	Modifies alternative splicing to increase SMN protein expression or reduce mutant HTT level	SMA, HD	Discontinued
PTC‐518	*HTT*	Induces PE inclusion to reduce mutant HTT level	HD	Phase 2 (NCT06254482)
PTC‐607	Undisclosed	Undisclosed	HD	NHV Phase 1 (ACTRN12622001534774)
SKY‐0515	*HTT/PMS1*	Induces PE inclusion to reduce both mutant HTT and PMS1 level	HD	Phase 1/2 (NCT06873334)
REM‐422	*MYB*	Induces PE inclusion to reduce MYB level	ACC, AML/MDS	Phase 1 (NCT06118086, NCT06297941)
RGT‐61159	*MYB*	Induces PE inclusion to reduce MYB level	ACC, AML	Phase 1 for ACC (NCT06462183), IND enabling for AML
ERX‐963	*DMPK*	Binds to r(CUG) repeats and rescues MBNL1	DM1	Phase 1 completed (NCT03959189) but without efficacy, now discontinued
Zotatifin	Eif4A/Mrna complex	Stabilizes incompetent RNA/Eif4A complex and interrupts translation initiation	Breast cancer	Phase 1/2 (NCT04092673)
RTX‐317	*HTT/PMS1*	Induces PE inclusion to reduce both mutant HTT and PMS1 level	HD	Preclinical
ARK‐175876	*MYC*	Binds to 5′‐UTR and inhibit translation	Oncology	Preclinical
ARK‐178164	*DMPK*	Binds to r(CUG) repeats and rescues MBNL1	DM1	Preclinical

In summary, this section highlights the diverse mechanisms by which bioactive small molecules target RNA to modulate gene expression, offering therapeutic potential across a wide range of diseases. Despite ongoing challenges in compound selectivity and clinical translation, the increasing number of preclinical and clinical candidates validates RNA as a viable therapeutic target. Future success in this field hinges on appropriate RNA target selection, robust mode‐of‐action confirmation, thorough off‐target evaluation, and structural studies of RNA‐small molecule binding to inform lead optimization.

## 4 Computer‐Aided Ligand Design

Computer‐aided design of RNA‐targeted small molecules faces challenges from the inherent structural flexibility and dynamic nature of RNA, which complicate accurate binding predictions and ligand optimization. Current computational approaches need to overcome limitations like sparse high‐resolution RNA–ligand complex data, inadequate chemical space exploration, and scoring function biases adapted from protein‐centric methods. Future progress hinges on integrating advanced computational techniques with experimental validation, expanding RNA‐specific chemical libraries, and refining machine learning models to improve predictive accuracy. Addressing these challenges will accelerate the discovery of RNA‐targeted therapeutics, offering new avenues for treating diseases with previously undruggable RNA targets. This section explores these hurdles and emerging strategies to advance the field.

### 4.1 Bioinformatics in Target Discovery

**FIGURE 3:**
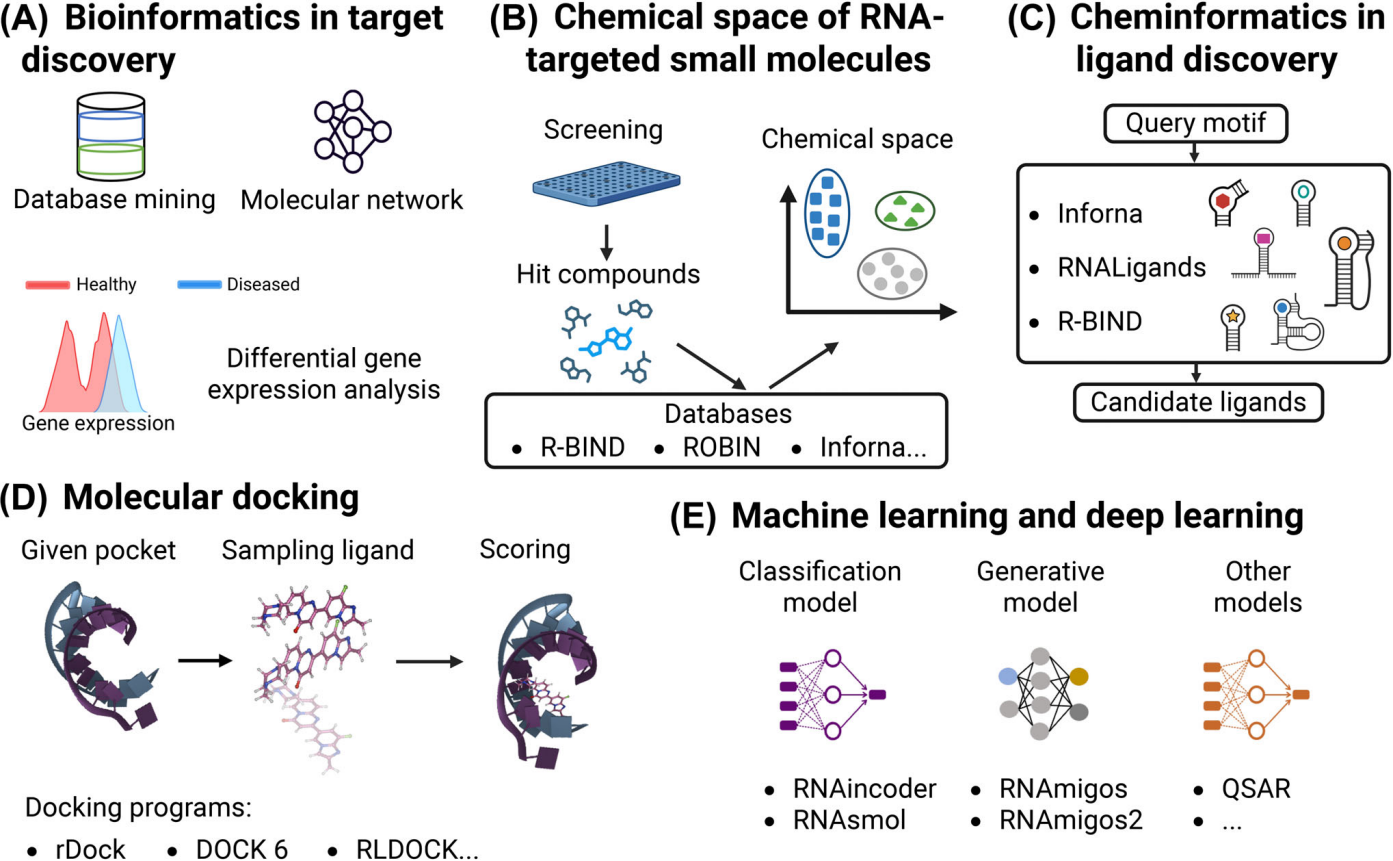
Computer‐aided ligand design. (A) There are many bioinformatics methods used in target discovery, including database mining, computational modelling of molecular networks, and differential gene expression analysis. (B) High‐throughput screening methods are used to identify target‐binding ligands, with results collected in databases such as R‐BIND and ROBIN, which help define the chemical space of RNA‐targeted small molecules. (C) Cheminformatics pipelines in ligand discovery. (D) The molecular docking involves the target pocket identification, ligand sampling, and postdocking scoring refinement. (E) Several machine learning and deep learning models are built for computer‐aided drug design, which can primarily be categorized into three classes: classification models, generative models, and other models. Created with BioRender.com.

Potential RNA targets are those found overexpressed in diseased cells and proved with definite functional roles in disease pathogenesis [19]. Akin to protein peers, RNA could form cleft‐like, druggable binding pockets, utilizing specific secondary motifs that are not fully base‐paired. The computational discovery of RNA targets involves the inference from functions and structures of RNAs. For mRNAs, in‐depth exploration of related targets involves integrating genomic, transcriptomic, and proteomic data to map out disease‐associated pathways. Techniques such as differential expression analysis and network‐based approaches help in pinpointing key mRNAs that play crucial roles in disease progression (Figure 3A). Furthermore, advancements in RNA‐seq and HTS technologies enable comprehensive profiling of mRNA expression patterns, facilitating the identification of novel therapeutic targets. For miRNAs, bioinformatics tools can predict target sites based on sequence complementarity and thermodynamic stability. Databases like TargetScan [168], miRDB [169], and miRTarBase [170] provide extensive information on experimentally validated and computationally predicted miRNA targets (Figure 3A). Structural insights gained from techniques like X‐ray crystallography and NMR spectroscopy further enhance the accuracy of miRNA target prediction. Computational methods, including machine learning models and motif‐based searches, are utilized to predict lncRNA interactions with other biomolecules. Correlation analyses with expression profiles and functional assays validate these predicted interactions, shedding light on the regulatory roles of RNAs in various biological processes and diseases. For example, circular RNA ciRS‐7 regulates the expression of genes associated with neurodegenerative diseases, such as Alzheimer's disease, by competitively binding with miR‐7. Bioinformatics analysis has predicted that miR‐7 is a target of ciRS‐7, along with its downstream regulated genes [171]. To summary, the computational discovery of RNA targets encompasses a multifaceted approach, integrating multiomics data, predictive modeling to identify and validate RNA molecules involved in disease mechanisms. This integrative strategy paves the way for developing RNA‐targeted therapies, offering new avenues for treating complex diseases.

### 4.2 Chemical Space of RNA‐Targeted Small Molecules

Experimental techniques such as the automated ligand identification system (ALIS) and SMM were applied to the screening of RNA‐small molecule interactions. These HTSs primarily identify potential ligands that bind to RNA molecules in specified chemical space [172] (Figure 3B), which is crucial for the discovery of RNA‐targeted therapeutics. For instance, Merck & Co. conducted an extensive ALIS screening across 42 RNA targets, analyzing the binding preferences of small molecules toward RNA targets with diverse secondary structures [173]. Additionally, Yazdani et al. [174] utilized SMM to screen 27 RNA targets and curated ROBIN dataset, then employed machine learning tools to analyze the physicochemical properties of small molecules that bind to RNA, leading to features that classify protein‐binders and RNA‐binders. Several comprehensive databases have been collected to curate and catalog experimentally determined interactions of bioactive small molecules and RNA targets, for example, R‐BIND [137] and ROBIN [174]. These databases are constructed based on the hypothesis of an RNA‐biased chemical space to uncover RNA‐privileged properties. They serve as valuable resources for identifying and developing successful RNA‐targeted probes and small molecules that modulate RNA function. However, the complex and dynamic nature of RNA–ligand interactions means that experimental results can vary significantly depending on factors such as buffer conditions, assay readout, test approaches, and other protocols used. This variability underscores the need for continued refinement to ensure the reliability of the defined RNA‐targeted chemical space.

### 4.3 Cheminformatics in Ligand Discovery

Developing computational prediction methods for the initial screening of candidate compounds can significantly reduce research and development costs. Currently, cheminformatic strategies applied to the study of RNA–ligand binding based on RNA structural motif libraries and data mining (Figure 3C). Researchers have proposed a prediction method based on an RNA structural motif library called Inforna [175]. This method leverages a two‐dimensional combinatory screening dataset, to predict the most suitable ligand against the specific RNA secondary structure by calculating its fitness score, which stores the selectivity and avidity profile of the small molecule. Similarly, RNALigands [176] performed data mining from R‐BIND, PDB, and Inforna to provide a comprehensive resource for the RNA‐targeting chemical space, which could inspire the selection of desired small molecule binding to the queried RNA secondary structure motif. Inforna 2.0 identifies RNA secondary structure and finds the corresponding ligand partners with fitness scores for a given motif. It is an updated version doubling the size of the database of Inforna, which has enabled the design of small molecules and modularly assembled compounds that modulate dysfunction of RNAs that are linked with human disease [131, 177]. Warner et al. [20] showed that RNA complexity in terms of the capacity for ligand‐binding could be characterized by its information content, whereas RNA motifs with high information content tend to have potential high‐specificity and high‐potency binding [178, 179]. This concept underscores the importance of understanding RNA's structural characteristics in drug design. Designing small molecules that specifically target RNA structures presents unique challenges compared with protein‐targeting drug design. In three‐dimensional structures, stacking between adjacent bases plays a crucial role in stabilizing RNA structures, whereas protein folding is predominantly driven by hydrophobic interactions. Correspondingly, in the interaction patterns with small molecules, the contribution of stacking is more significant, and the contribution of hydrophobic interactions is less than in proteins [180, 181]. These cheminformatic methods have been primarily applied to short RNA two‐dimensional structure motifs, and its effectiveness for complex RNA structures has not been fully validated.

### 4.4 Molecular Docking: Reprogramming Docking Tuning from Proteins and RNA‐Biased Docking Tools

Computational modeling of RNA‐small molecule interactions has allowed fast and accurate VS to accelerate selection of potent small molecules as drug candidates. RNA–ligand docking remains a formidable challenge because (1) RNA is highly charged and often requires the participation of metal ions and water to stabilize the binding pocket conformation and mediate RNA–ligand interactions; (2) RNA molecules are intrinsically of high flexibility; (3) we have a paucity of experimentally determined structures for RNA‐only and RNA–ligand complex structures. The docking pipeline (Figure 3D) usually includes three components: (1) identifying druggable RNA targets and prospective binding sites; (2) efficient sampling of ligand binding modes; and (3) accurate scoring functions for RNA–ligand docking [182].

In the context of molecular docking, a prevalent practice involves docking small molecules onto specific regions of interest within RNA. The identification of these specific regions or potential binding pockets can be accomplished through multiple approaches. The most reliable approach stems from domain knowledge regarding the functional sites of RNA that engage in crucial interactions with other biomolecules. By directly targeting such functional sites, one can potentially identify not only an RNA binder but also one that is functionally relevant. Alternatively, the determination of docking pockets can be guided by various pocket definition algorithms. For instance, DOCK 6 selects the docking pockets from a negative image of the receptor surface, where each cavity is characterized by a set of overlapping spheres [183]. rDock applies either a two‐sphere method or the reference ligand method to define the docking site [184]. Other methods find docking pockets by estimating the overall probe‐pocket interaction energies by rolling a small‐molecule probe over the surface of RNA [184]. For example, PocketFinder in ICM uses Lennard‐Jones potential to describe the interactions between the probe and receptor atoms [185]. While AutoLigand in AutoDock uses a similar approach, with an extra iterative step to identify the optimal binding sites and account for connections between neighboring pockets [186]. Both rDock and AutoDock can be used for local docking, where the position of a reference ligand is provided, and for blind docking, which reveals potential binding pockets without prior knowledge of the binding site. In contrast, DOCK 6 and ICM are restricted to local docking. Machine learning models, such as Rsite and Rsite2, treat RNA–ligand interactions as a network of contacting atoms and predict functional sites based on the clustering of nts [187, 188]. RBind transforms an RNA structure into a graph, and predict the functional sites as regions formed by nts of the maximum closeness [189, 190]. Additionally, RNAsite uses a random forest‐based model along with sequence‐based and/or structure‐based descriptors to predict whether a given nt belongs to the functional sites [191].

Sampling ligand binding modes includes sampling ligand conformations and RNA conformations. The multiconformer docking algorithm prepares a conformational ensemble for a ligand and performs rigid docking for each ligand conformer against the same target. RLDOCK is a recently developed docking model for flexible ligands using a multiconformer approach [192, 193]. DOCK6 adopts incremental construction strategy for ligand conformational sampling. It anchors rigid fragments through geometric matching and then incrementally builds the ligand structure [183]. AutoDock Vina adopts hybrid approach to sample ligand conformations. It uses MC for global optimization of binding modes and Broyden–Fletcher–Goldfarb–Shanno algorithm for local optimization of binding modes [194]. ICM and rDock adopt similar hybrid approach by employing MC coupled with simulated annealing [195, 196]. For RNA conformation sampling, Glide and GOLD allow users to decrease the energy penalties for steric clashes thus tolerating some degree of overlap between RNA and ligand for soft docking [197, 198]. Docking a given ligand into an ensemble of RNA conformations or an ensemble‐averaged RNA conformation also accounts for the flexibility of RNA molecules. Such ensemble docking‐based VS with the ICM docking model for HIV‐TAR has predicted a TAR‐targeting compound with high specificity [70, 199]. MORDOR samples conformation and show ligand‐induced conformational changes by applying an RMSD penalty term to the conventional potential energy and run MD simulation [200]. Methods based on elastic potential grids, which originally proposed for modeling protein flexibility [201], can be used to better account for the flexibility in RNA structure during docking [202]. Such methods first calculate a 3D grid of the potential field of the initial RNA conformation using DrugScore^RNA^ and uses AutoDock as a docking engine along with this potential grid for docking [194, 202, 203].

Selecting a native ligand binding pose from an ensemble of candidates requires a reliable scoring function. Various scoring methods have been proposed, including physics‐based, knowledge‐based and machine learning‐based approaches. Physical‐based methods, such as Generalized‐Born [204] and Poisson‐Boltzmann [205] implicit solvent models, when combined with the AMBER force fields [206], can be used in DOCK6 to provide an effective energy model for the RNA–ligand docking system [183]. Additionally, iMDLScore1 and iMDLScore2 optimize RNA‐specific semi‐empirical free energy functions as scoring functions [207]. Several RNA–ligand docking software such as rDock also has their respective empirical scoring functions [184]. Knowledge‐based scoring functions, used in Drugscore^RNA210^ and LigandRNA [208], are derived from the statistical potential approach which applies the inverse Boltzmann law to extract energy‐like potentials from interacting pairs. RNA‐tailored machine learning scoring method is an emerging scoring approach. RNAmigos is a machine‐learning model designed for the prediction of RNA‐small molecule interactions [209]. In RNAmigos, the base‐pairing network around the binding site is simplified as a connected 2D graph with vertices and edges, where a nt is represented by a vertex and backbone connectivity and base‐pairing are represented by the different types of edges. The base‐pairing interactions encoded in the 2D graph provide a signature for predicting the fingerprint of the small molecule that will most likely bind to the site. RNAPosers uses pose fingerprints to describe RNA–ligand interactions [210]. By training classifiers to distinguish native from non‐native conformations based on these fingerprints, the method achieves 80% accuracy in identifying RNA–ligand poses within 2.5 Å of the true structure, significantly outperforming traditional docking scoring methods. AnnapuRNA is a knowledge‐based scoring function that uses machine learning techniques including k‐nearest neighbors (k‐NNs) and deep learning to predict RNA‐small molecule binding mode [211]. Trained on experimental RNA–ligand complex structures, AnnapuRNA distinguishes between observed and theoretical interactions, optimizing parameters like distance thresholds and angles. Evaluated on 33 RNA–ligand complexes, it demonstrates superior performance in identifying binding modes, particularly for challenging cases. RSAPred is a machine learning model using multivariate linear regression to predict RNA‐small molecule binding affinity for six RNA subtypes, achieving high accuracy with a mean Pearson correlation coefficient of 0.83 and an average absolute error of 0.66 in jack‐knife testing [212]. The model is available via the RSAPred web server. RLaffinity couples a 3D convolutional neural network (3D‐CNN) with a contrastive learning‐based self‐supervised pretraining model for RNA‐small molecule binding affinity prediction [213]. It uses 3D‐CNN to extract both global pocket information and local neighbor nt information within RNAs and further enhances its predictive performance by using the pretraining model.

Kallert et al. [214] introduce several challenges encountered during protein‐based VS for RNA–ligand docking along with potential solutions. While protein‐based docking tools demonstrate similar performance to RNA‐specific algorithms like rDOCK, unique challenges persist. One pitfall lies in the protomer and tautomer selection, which requires careful consideration of ligand conformations and ionization states to accurately mimic binding interactions. Target dynamics and explicit solvent modeling also pose challenges, necessitating the use of flexible docking protocols and solvent models optimized for RNA targets. To address these, the study from Kallert et al. suggests incorporating RNA‐specific features into scoring functions and optimizing protocols to handle the high flexibility and negatively charged backbone of RNA. Additionally, the study emphasizes the importance of selecting VS hits not solely based on docking scores but also by evaluating their ability to mimic key interactions of the native ligand, thereby enhancing the chances of identifying novel active binders.

Several studies have benchmarked different docking programs and characterized their performance profiles. Jiang et al. [215] collected a large dataset of 800 noncovalent nucleic acid (NA)–ligand complexes with clearly identified ligands. Based on this dataset, they systematically evaluated eight commonly used docking programs including six for protein–ligand docking (LeDock [216], Surflex‐Dock [217], DOCK6 [183], AutoDock [186], AutoDock Vina [194], and PLANTS [218]) and two for NA–ligand docking (rDock [184] and RLDOCK [192], [193]). PLANTS, rDock and LeDock showed highest performance in binding pose prediction. However, few programs were successful in binding affinity prediction, with PLANTS performed the best (*R*_p_ = −0.461). Further comparisons with NLDock [219] demonstrated that PLANTS and LeDock outperformed NLDock across all tested datasets. The study also underscored the challenges of RNA docking due to structural flexibility and highlighted the need for improved scoring functions and algorithms tailored to NA systems, as current methods lag behind protein–ligand docking in predictive accuracy. Agarwal et al. [220] tested the binding pose prediction accuracy of AutoDock4 [221, 222], AutoDock Vina and rDock on 173 RNA‐small molecule crystal structures, It compares their success rates in generating ligand poses with RMSD thresholds of 1.5–2.5 Å, revealing that rDock performs best when a bound ligand's crystal structure is available to define the docking search space (48% success rate for top poses, 63% for best of top five poses), while AutoDock Vina achieves comparable results under similar conditions but struggles in scenarios without prior ligand‐bound structures (both programs exhibit low success rates of ∼27%). The study also identifies AutoDock Vina's bias toward ligands with specific physicochemical properties (e.g., lower molecular weight, fewer hydrogen bonds), whereas rDock maintains consistent performance across diverse ligand features. Additionally, parameter tuning (e.g., box size, exhaustiveness) shows limited improvement in docking accuracy, underscoring intrinsic challenges in RNA docking due to RNA's structural flexibility and unique binding environments.

### 4.5 Machine Learning and Deep‐Learning Models in Ligand Discovery

In recent years, with the rapid development of artificial intelligence, many computational methods have attempted to integrate the latest deep learning technologies to study small molecule interactions with RNAs, overcoming limitations of traditional methods such as molecular docking. In addition to these advancements, there have been attempts to build generative models to study the design of small molecule drugs targeting RNA (Figure 3E). RNAsmol [223] and RNAincoder [224] attempted to use data augmentation and feature engineering strategies to predict the interaction between RNAs and small molecules. Furthermore, there have been quantitative structure–activity relationship model designed for RNAs to predict both thermodynamic and kinetic binding parameters of small molecules [225, 226]. RNAmigos and RNAmigos2 models [209, 227] represent RNA's three‐dimensional structure as a graph containing multiple edge information and optimizes the relational graph neural network to predict the binding preferences between the RNA and small‐molecule drugs. Moreover, molecular structural fingerprints [174] and structural interaction fingerprints [228], combined with machine learning techniques, have been used to develop predictive models for ligand binding to RNA and to elucidate the interactions between small molecules and RNA. Despite of these accomplishments, the rational design of small molecules targeting RNA currently confronts with numerous challenges. First, the highly complex nature of RNA structures and limitations in experimental techniques have resulted in a relatively small number of nonredundant high‐resolution 3D structures for known RNA, numbering only in the hundreds. This number is significantly less than the known protein structures, with only 8331 RNA‐containing structures in the RCSB PDB, accounting for about 3.6% of the total number of structures (230,083 as of January 2025). Additionally, chemical space of small molecules binding to RNA has not been thoroughly explored. Existing data primarily focus on screening experiments for relatively short RNA fragments, and the available data are sparse and inadequate. Consequently, utilizing deep learning models to assist in targeting RNA drug research imposes high demands on the model's applicability and generalization capabilities.

Computer‐aided ligand design for RNA targets struggles with RNA's structural flexibility, sparse high‐quality datasets, and vast chemical diversity, limiting docking accuracy and predictive power of machine learning models. While MD simulations have emerged as a powerful tool to address RNA flexibility by capturing conformational dynamics and refining binding mechanisms, their utility is constrained by computational costs and force field limitations. Overcoming these challenges requires improved computational models, expanded RNA‐specific chemical libraries, and better integration of experimental data (e.g., NMR, small‐angle X‐ray scattering) to enhance the accuracy of RNA structural ensembles. Furthermore, integrating multiscale computational approaches, such as MD‐enhanced VS or machine learning‐guided simulations, with experimental validation demands substantial resources and interdisciplinary collaboration. Looking forward, future directions in this field should focus on refining RNA force fields, optimizing MD sampling techniques to enhance the accuracy of RNA structural models, expanding chemical libraries with RNA‐compatible scaffolds, and developing more sophisticated machine learning frameworks tailored to the unique challenges of RNA targeting. Advances in high‐performance computing, artificial intelligence, and collaborative platforms will be crucial in overcoming these obstacles, ultimately enabling the discovery of novel RNA‐targeted therapeutics with improved efficacy and specificity.

## 5 Screening and Hit Identification for RNA Binders

To date, HTS methodologies have been the predominant approach for discovering RNA‐targeted small molecules. In conjunction with downstream target validation protocols, different screening methodologies have proven o be effective. Challenges persist due to RNA's structural complexity and dynamic nature, necessitating innovative screening and library design strategies. This section explores cutting‐edge approaches, including focused screenings (FcSs), DELs, SMMs, and fragment‐based drug discovery (FBDD), that leverage RNA‐biased chemical spaces and high‐throughput technologies to identify and optimize RNA binders. By addressing limitations such as false positives in DELs, immobilized ligand constraints in SMMs, and weak affinities in FBDD hits, these methodologies aim to bridge the gap between RNA's intricate biology and the development of selective therapeutics.

### 5.1 FcSs and Library Curation

The intrinsic chemical nature of RNA molecules suggests a corresponding chemical space within which its bound ligands should reside. A recent analysis of privileged interactions underlying small molecule–RNA recognition, based on data from the PDB, revealed that RNA recognition is primarily driven by stacking and hydrogen bonding interactions, in contrast to protein recognition, which is predominantly governed by hydrophobic effects [180]. This divergence in interaction forces results in markedly distinct chemical spaces for RNA‐binders versus protein binders, as elucidated by the Hargrove lab and their cheminformatic analyses utilizing R‐BIND [229], a comprehensive database of reported RNA‐targeted bioactive small molecules.

**FIGURE 4:**
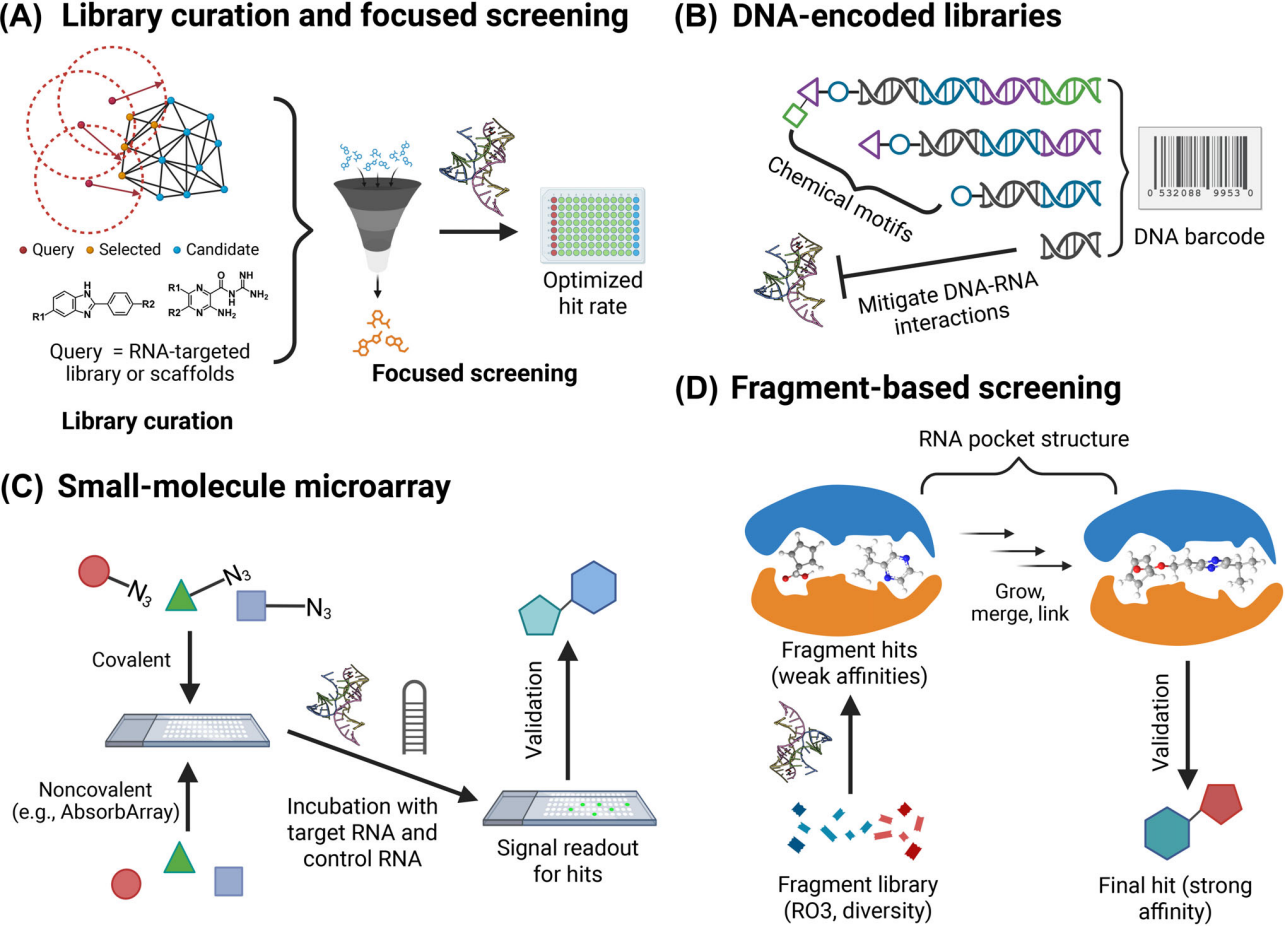
Methods that generate hit compounds against RNA as the target. (A) Focused screening have afforded most of hit compounds targeting RNA among HTS campaigns, which usually starts with a library curation to give a curated screening set for improved hit rates. (B) DELs are an innovative approach in hit identification that combines combinatorial chemistry with high‐throughput DNA sequencing, while mitigating DNA–RNA interactions is essential to avoid false positives. (C) Small‐molecule microarrays involve the covalent immobilization or noncovalent incubation (e.g., AbsorbArray) of a diverse array of small molecules on a solid surface, such as glass slides or microplate wells. The screening could be done in an efficient fashion with thousands of compounds being tested simultaneously. (D) Fragment‐based screening allows weak binders to be grew, merged, and linked to a larger hit molecule, usually guided by the high‐resolution RNA–ligand complex structure. The method could effectively evaluate the chemical space using a relatively small‐size screening library containing small fragments. Created with BioRender.com.

FcS methodologies employ curated libraries predicated on the existence of a privileged RNA‐biased chemical space that favors RNA binding (Figure 4A). The enhanced hit rates observed in several FcS approaches provide compelling evidence for the efficacy of this strategy in targeting RNA, mirroring its established success with protein targets [230]. Kutchukian et al. [173] conducted an extensive study utilizing the ALIS to screen 42 distinct RNA targets against a library of approximately 50,000 chemically diverse, drug‐like small molecules. The study employed machine learning techniques, specifically naïve Bayesian models, to analyze the physicochemical properties and chemical features of identified binders. This analysis facilitated the construction of an RNA‐focused library comprising around 3700 small molecules with an increased propensity for binding to the RNA target set, demonstrating a significantly improved average hit rate of 0.32% compared with 0.04% without library curation. Hargrove et al. [231] developed a FcS library, the Duke RNA‐Targeted Library (DRTL), based on the physicochemical and structural properties of R‐BIND small molecules. Through the analysis of over 2 million commercially available small molecules using a k‐NN algorithm, DRTL was curated to include 804 small molecules from the initial library, representing one of the largest academic RNA‐focused libraries to date. The compounds were evaluated in vitro against a panel of four RNA targets using optimized FID assays. The screening identified multiple small‐molecule hits, including novel scaffolds for RNA, with hit rates ranging from 0.87 to 3.1%, comparable to other RNA‐focused libraries. The accumulating results from various screening campaigns have substantiated the importance of library curation for the effective identification of initial hit compounds with novel scaffolds and chemotypes in RNA‐targeted drug discovery.

### 5.2 DNA‐Encoded Libraries

DELs have emerged as a powerful tool in modern drug discovery, offering an efficient means to evaluate vast numbers of screening candidates. DELs typically comprise a diverse pool of compounds synthesized through fundamental chemical reactions, including click chemistry, peptide bond formation, reductive amination, and Suzuki coupling [232]. Each compound within a DEL is uniquely identified by a DNA barcode that encodes its structural information, which can be subsequently decoded through next‐generation sequencing and computational analysis.

While DELs have demonstrated significant success in protein‐binder discovery, their potential for RNA‐binder identification has only recently begun to be explored. A primary challenge in applying DELs to RNA targets is the propensity for DNA tags to bind to the RNA target, potentially leading to false positives (Figure 4B). This issue can be mitigated by reducing the equivalency of DNA tags in solid‐phase DEL systems and/or incorporating base‐paired controls and RNA competitors. Furthermore, the diversity of chemical motifs incorporated into the test candidates may be constrained by the limited repertoire of chemical reactions that exhibit compatibility with RNA molecules. Stringent control of building block purity is imperative to mitigate the occurrence of false positives and low‐quality hits [233]. In practical applications, rigorous re‐synthesis and comprehensive experimental validation of selected hits are essential steps for hit validations.

Dou et al. [10] encountered DNA tag: RNA hybridization problem when employing DEL selection against the HIV‐1 TAR, observing that the majority of signals were attributable to false‐positive DNA–RNA binding. In response, they developed an optimized selection strategy utilizing RNA patches and competitive elution to minimize undesired DNA binding, followed by k‐mer analysis and motif searching to differentiate false‐positive signals. This refined approach yielded significantly improved results when applied to the Escherichia coli FMN Riboswitch, with enriched compounds demonstrating double‐digit nanomolar binding affinity and comparable potency in functional FMN competition assays.

Recent advancements by Disney et al. [234] have further enhanced DEL technology by integrating 2DCS with solid‐phase DEL in a massively parallel screening pipeline. This innovative approach employs fluorescence‐activated cell sorting to identify DEL beads that specifically bind to RNA folds, facilitating the isolation of selective small‐molecule RNA ligands. By mimicking nature's highly parallel ligand‐target search paradigm, researchers were able to evaluate the affinity landscapes between 4096 RNA folds and 73,728 small molecules, identifying numerous bona fide RNA–ligand pairs. This methodology led to the discovery of a potent small‐molecule ligand that selectively bound to a 5′GAG/3′CCC internal loop present in primary miRNA‐27a (pri‐miR‐27a), an oncogenic precursor of miRNA‐27a. Subsequent studies validated the hit molecule's ability to disrupt the biogenesis of mature miRNA‐27a and demonstrated its efficacy in inhibiting migration and invasion of triple‐negative breast cancer cells.

These findings underscore the utility of DELs in identifying high‐affinity and selective target–ligand interactions, potentially facilitating the development of therapeutic agents capable of modulating disease‐associated phenotypes.

### 5.3 Small‐Molecule Microarrays

SMMs represent a HTS methodology that facilitates the simultaneous evaluation of numerous interactions, particularly advantageous for identifying RNA binders within diverse chemical libraries. The SMM approach involves the immobilization of a ligand library on a functionalized solid surface, typically glass slides, via specific chemical linkages. The surface is often modified with reactive groups, such as isocyanates, to ensure covalent attachment of the small molecules. The microarray undergoes incubation with fluorescently labeled RNA(s) of interest, enabling the detection of RNA‐small molecule interactions. Subsequent washing steps are crucial for discriminating between specific and nonspecific binding. Fluorescence scanning of the microarray allows for the visualization of bound RNA, with signal intensity at each spot indicative of interaction strength [235].

A primary advantage of SMM in early hit identification lies in its capacity to screen large libraries with minimal material consumption. However, a common limitation is the evaluation of RNA binding in an immobilized ligand state rather than in free solution. To address this constraint, Disney et al. [236] developed AbsorbArray, a noncovalent variant of SMM (Figure 4C). This approach involves absorbing the small‐molecule library onto hydrated agarose‐coated microarray surfaces, followed by incubation with ^32^P‐radiolabeled RNA library for 2DCS. Subsequent phosphorescence imaging, isolation of radioactive spots, and sequencing analysis facilitate hit compound identification.

The Disney group have applied AbsorbArray to screen a combinatorial compound library against an RNA library with randomized internal loop patterns, incorporating excess oligonucleotides to mitigate undesirable binding. This screening strategy led to the discovery of a potent miRNA‐21 precursor binder capable of modulating mature miRNA‐21 biogenesis by selectively targeting the Dicer processing site [236].

Given their high‐throughput nature and robust quality control, SMMs have been employed to screen various commercial and customized libraries, generating valuable RNA–ligand binding data. Notable examples include the Inforna database compiled by Disney's laboratory, which can be integrated with structure–activity relationship through sequencing (StARTS) to score RNA motif–ligand pairs [131]. The resultant fitness scores can guide the selection of optimal RNA motifs for targeting by specific ligands. Screening campaigns conducted by Schneckloth et al. [174] have also contributed significantly to the documentation of RNA–ligand paired data, including ROBIN, one of the most extensive public datasets to date.

Consequently, SMMs have proven particularly effective in evaluating RNA–ligand binding, potentially leading to the development of therapeutic agents or providing insights for rational ligand design.

### 5.4 Fragment‐Based Drug Discovery

FBDD represents a versatile approach to explore large chemical space in relation to RNA targets, utilizing comparatively small, RNA‐biased fragments. The fundamental premise of FBDD posits that authentic hit molecules exhibit binding affinity and specificity toward the target via specific functional moieties. Consequently, the direct application of small fragments containing these crucial functional groups should elicit detectable binding signals through biophysical assays or phenotypic readouts, notwithstanding potentially weak binding affinities. The screening library typically adheres to the “Rule of 3” criteria, stipulating that compounds possess molecular weight below 300, clogP values below 3, and less than three hydrogen bond donors/acceptors (Figure 4D) [237]. FBDD demonstrates a marked advantage over traditional HTS in terms of efficiency, as the screening of thousands of related fragments is sufficient to encompass the chemical space occupied by millions of full‐sized compounds. Moreover, the utilization of small fragments mitigates the incidence of false positives often associated with poor solubility and nonspecific binding of larger molecules. However, FBDD is primarily considered an initial screening method in the early stages of hit identification. Hit validation necessitates the implementation of at least one orthogonal method to corroborate the credibility of identified hits. The fragments identified through this process typically exhibit low drug‐likeness and weak binding affinities, necessitating subsequent elaborate expansion steps, including growth, merging, and linking, to enhance their binding profiles (Figure 4D) [238].

NMR represents a widely employed methodology for fragment screening, permitting the simultaneous evaluation of multiple ligands, provided their ^1^H resonances exhibit minimal overlap. The binding of ligands can be readily detected through ligand‐induced chemical shifts of RNA imino protons. In a recent study, Schwalbe et al. [239] conducted an NMR screening campaign targeting the 5′‐UTR of the SARS‐CoV‐2 virus genome, utilizing two distinct fragment libraries: one RNA‐dedicated (DRTL‐F) and another protein‐biased (DSI‐PL). This investigation explored the feasibility of dual ^1^H and ^19^F signal detection in NMR experiments, potentially reducing measurement time and enhancing signal sensitivity. Hit criteria were established based on significant chemical shift and signal intensity changes in ^1^H 1D, ^19^F 1D, and ^19^F T_2_‐CPMG experiments. The study identified several mid to low micromolar binders against SARS‐CoV‐2 5′‐UTR stem loops, particularly from the RNA‐dedicated DRTL‐F library, underscoring the critical role of library design in FBDD. Alternative screening methodologies, including mass spectrometry, surface plasmon resonance, fluorescence displacement assays, and virtual screens, offer varying degrees of throughput and material costs, allowing flexible adoption in initial screenings or as orthogonal hit validation methods [238].

Following initial hit identification, optimization processes are essential to enhance compound binding affinity and selectivity. FBDD employs three primary strategies for optimizing initial fragment hits into lead compounds: fragment growing, merging, and linking. The fragment growing strategy involves the incremental addition of functional groups to the initial fragment, aiming to establish additional binding interactions with the target RNA. This approach necessitates detailed structural information of the RNA–fragment complex. During the growing process, ligand efficiency and synthetic feasibility must be considered to maintain the functional core structure within the correct mode of action. Fragment linking combines two or more fragments that bind to adjacent pockets of the target, requiring precise structural knowledge of fragment binding positions for accurate molecular design, including linker length and orientation. This strategy can lead to significant potency increases due to the additive effect of multiple binding interactions. Fragment merging integrates structural features from multiple overlapping fragments into a single molecule, particularly useful when fragments bind in the same or overlapping pockets. This approach often results in compounds that maintain key interactions of both parent fragments, potentially leading to improved potency and reduced molecular weight compared with linking strategies.

In conclusion, advances in RNA‐targeted screening technologies, from curated focused libraries to DELs and FBDD, have significantly enhanced the identification of RNA‐binding small molecules, yet challenges in specificity, affinity, and scalability persist. Future progress will depend on refining these methodologies, such as minimizing hybridization artifacts in DELs and integrating computational tools with experimental validation, to expand the druggable RNA landscape. As these strategies mature, they hold immense potential to unlock novel therapeutic avenues for diseases driven by previously undruggable RNA targets, marking a transformative phase in RNA‐centric drug discovery.

## 6 Small Molecules that Degrade RNA

Extensive research of RNA‐targeted chemical probes has reached a consensus that the binding of small‐molecule probes to the target RNA sequences does not always bring out biological effects as expected. This phenomenon can be attributed to two primary factors: first, the propensity for off‐target binding to nonfunctional sites, and second, the inherent structural plasticity and rapid cellular turnover of RNA molecules, which may result in suboptimal or insensitive responses to small molecule binding events. When the functional site of an RNA molecule has not been identified or is difficult to target directly, promoting its degradation offers an alternative approach to modulating RNA function. This strategy can be particularly useful for targeting disease‐associated RNAs or for studying RNA function in cellular processes. Several innovative techniques have been developed to achieve targeted RNA degradation, including small‐molecule direct degraders, RiboTACs, and proximity‐induced NA degraders (PINAD) [240, 241].

### 6.1 Small‐Molecule Direct Degraders

A diverse array of small‐molecule compounds, ranging from natural products to organic–metal complexes, have been identified as direct RNA degraders. These compounds typically induce RNA degradation by producing highly reactive hydroxyl radicals, followed by hydrogen atom abstraction from the RNA structure. While these molecules offer a direct approach to modulating RNA stability, their practical application for targeted RNA degradation in cellular environments is limited by insufficient selectivity and a tendency to also degrade DNA.

**FIGURE 5:**
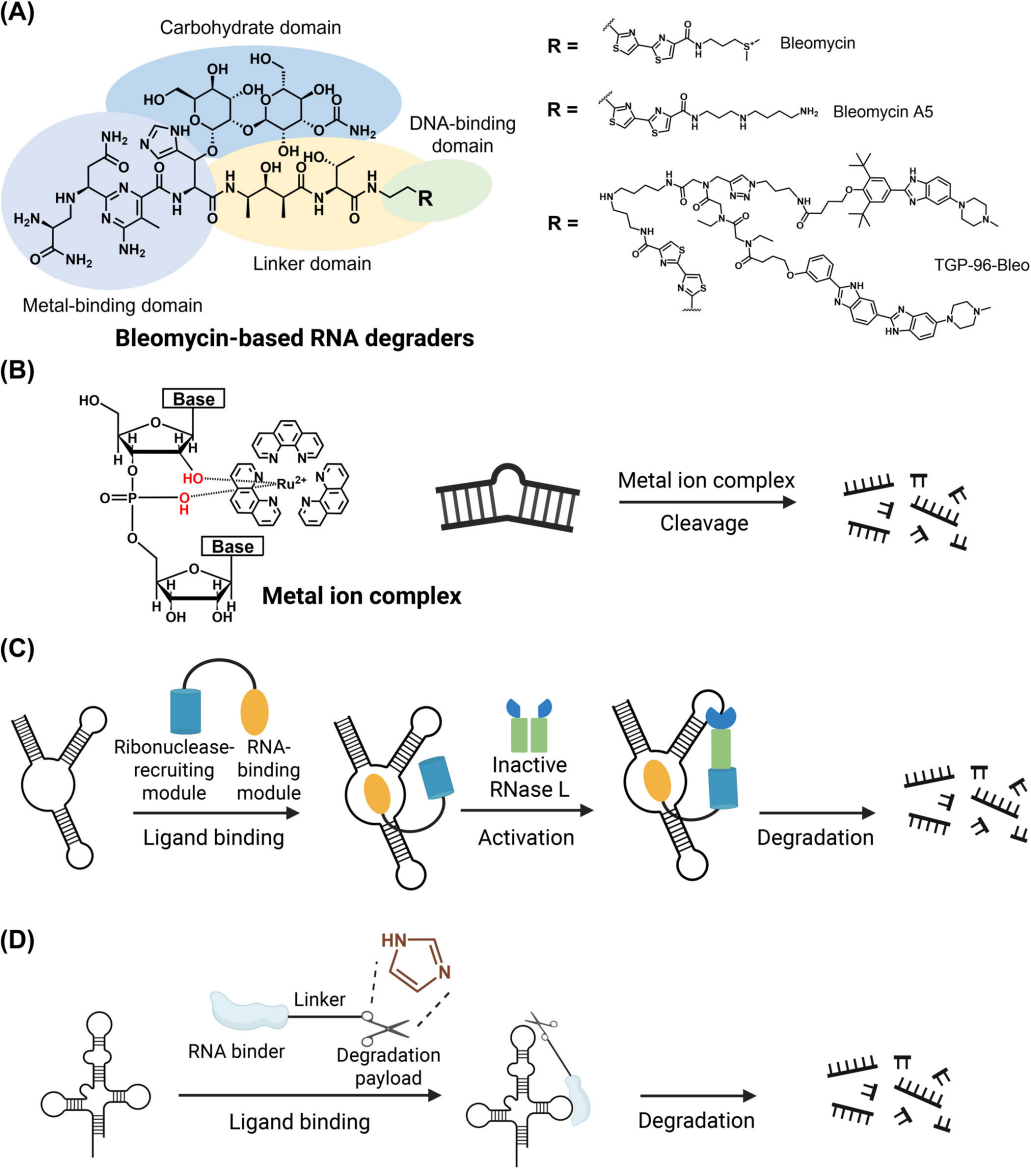
Small molecules that degrade RNA. (A) Schematic representation of engineered bleomycin‐based RNA degraders. The figure illustrates the structures of TGP‐96‐bleo and Cugamycin. TGP‐96‐bleo is created by conjugating Targaprimir‐96 (TGP‐96) with bleomycin A5, resulting in selective degradation of pri‐miRNA‐96 with reduced DNA cleavage activity. Cugamycin, formed by conjugating a small molecule targeting specific RNA motifs with bleomycin A5, preferentially cleaves hairpin RNAs with AU‐rich sequences. (B) Mechanism of RNA cleavage promoted by metal ion complexes. Metal ions such as Zn^2^⁺, Cu^2^⁺, and Mg^2^⁺ interact with the phosphate groups and 2′‐hydroxyl groups of the RNA backbone. This interaction facilitates the hydrolysis of phosphodiester bonds, leading to RNA strand cleavage. (C) Schematic representation of RiboTACs. It is comprised of an RNA‐binding module and a ribonuclease‐recruiting module that interconnected via a linker and can enhance RNA degradation by selectively binding to target RNA and recruiting RNase L. (D) Schematic representation of the proximity‐induced nucleic acid degrader (PINAD) featuring an RNA binding moiety linked to an imidazole payload for targeted RNA degradation. Created with BioRender.com.

Bleomycin is a well‐known anticancer agent that has been utilized as an effective RNA degrader in many studies. Structurally, bleomycin consists of four distinct domains: a metal‐binding domain, a carbohydrate domain, a linker domain and a DNA‐binding domain, each contributing to its overall function [242]. By engineering the DNA‐binding domain, we can develop a series of small molecules derived from bleomycin that exhibit selective RNA cleavage capabilities. These molecules incorporate an RNA‐binding module to their structures that facilitates the specific recognition of the target RNA, bringing bleomycin in close proximity for direct cleavage (Figure 5A). For instance, in a previous study, the compound Targaprimir‐96 (TGP‐96) was identified as an inhibitor of pri‐miRNA‐96 biogenesis. This inhibition is achieved through the compound's binding to the Drosha processing site [243]. By conjugating TGP‐96 with the free amine of bleomycin A5 through amide linkage, TGP‐96 was converted into a bleomycin degrader (TGP‐96‐bleo) (Figure 5A) [244]. Studies have demonstrated that TGP‐96‐bleo exhibited highly specific degradation of pri‐miRNA‐96 while simultaneously displaying diminished DNA cleavage activity in vitro and reduced DNA damage in cellular environments [243, 244]. Furthermore, Cugamycin, developed through the conjugation of a small molecule designed to selectively target a specific RNA motif with bleomycin A5, has shown a distinct preference for cleaving hairpin RNAs characterized by long AU‐rich sequences (Figure 5A) [245]. Compared with complementary locked NAs that degrade all tested short r(CUG) repeat‐containing mRNAs, Cugamycin specifically targets and degrades DMPK mRNA [246, 247]. In the DM1 mouse model, Cugamycin successfully degraded approximately 40% of mRNAs containing expanded r(CUG) sequences, restoring 97% of the splicing defects to patterns observed in wild‐type mice [247]. Subsequent research has optimized both the RNA‐binding and bleomycin moieties of Cugamycin, leading to the new conjugate with enhanced efficacy in patient‐derived myotubes from individuals with DM1 [248, 249].

Moreover, metal ion complexes, such as tris(1,10‐phenanthroline) ruthenium (II) complexes, have extensive applications in RNA cleavage [250, 251]. They often contain Zn^2+^, Cu^2+^, Mg^2+^, or other metal ions, which can interact with the phosphate groups and 2'‐hydroxyl groups in the RNA backbone, thereby promoting the cleavage of phosphodiester bonds (Figure 5B) [252, 253]. Metal ion complexes can bind with different ligands to achieve selective RNA cleavage. For instance, artificial ribonucleases, which couple metal ion complexes to oligonucleotides that guide selective cleavage, could hydrolyze RNA phosphodiester bonds in a sequence‐specific manner [254].

Collectively, these promising results underscore the potential and feasibility of utilizing small‐molecule direct degraders as therapeutic agents for the treatment of RNA‐related diseases. Nonetheless, the pharmacochemical optimization of Bleomycin Conjugates remains relatively limited, and the development of large‐scale manufacturing processes for different bleomycin domains poses significant challenges. Regarding metal ion complexes, further optimization of metal ion catalysts is needed to enhance their stability and selectivity within biological systems [254]. Additionally, incorporating other chemical modification strategies could improve their biocompatibility and specificity. In conclusion, small molecules hold substantial potential as RNA degraders. Subsequent optimization strategies may enable these molecules to exhibit even more efficacious outcomes.

### 6.2 Ribonuclease Targeting Chimeras

Proteolysis‐targeting chimeras (PROTACs) represent a category of bifunctional molecular constructs comprising two key elements: a protein‐binding domain and an E3 ligase recruitment domain, which are interconnected by a linker [255, 256]. PROTACs bind to a target protein with the protein‐binding moiety and recruit E3 ubiquitin ligase in situ, leading to the target protein degradation. Similar to PROTACs, RiboTACs are bifunctional molecules engineered to facilitate targeted RNA degradation. RiboTACs comprise an RNA‐binding module and a ribonuclease‐recruiting module that interconnected via a linker (Figure 5C). These molecules promote RNA degradation by selectively binding to the target RNA and bringing activated ribonuclease in close promixity [240]. RiboTACs frequently employ the ubiquitous endogenous ribonuclease RNase L to facilitate target RNA degradation. RNase L, a key component of the innate immune response against viral infections, transitions from an inactive monomeric state to an activated dimeric form to initiate RNA cleavage. Researchers have identified various chemical compounds, such as 2′‐5′‐oligoadenylates (2′‐5′poly(A)), that effectively activate RNase L. Recent advances in RNA degradation strategies have focused on two critical areas: the development of highly selective RNA‐binding molecules and the design of small molecules capable of efficiently activating and recruiting RNase L for localized target degradation.

For instance, an RNA binding module that selectively recognizes the Drosha site of pri‐miR‐96 or the Dicer site of pre‐miR‐210 is coupled to 2′‐5′A_4_ to induce selective cleavage in cells [257, 258]. Subsequently, a small molecule with higher drug likeness replaced the 2′‐5′A_4_ as the recruiting agent, thereby developing a RiboTAC that targets pre‐miR‐21 for degradation [259, 260]. The results showed that the bifunctional molecule inhibited breast cancer metastasis in a mouse model and was more selective than the binding molecule alone. In addition, known drugs with canonical activity can be reprogrammed to selectively affect RNA by converting to the RiboTAC. Dovitinib is a receptor tyrosine kinase inhibitor that also binds to the Dicer processing site of pre‐miR‐21. By conjugating Dovitinib to a RNase L recruiter module, Dovitinib‐RiboTAC was generated and found to have the ability to reduce the expression level of mature miR‐21 [261]. In the context of ALS and frontotemporal dementia (FTD) research, Disney et al. [262] have engineered and refined a RiboTAC specifically designed to target and degrade the pathogenic RNA expansion sequence r(G_4_C_2_)exp^exp^. Subsequent validation through both cellular assays and in vivo mouse models has demonstrated the compound's efficacy in reducing levels of r(G_4_C_2_)^exp^ RNA and its associated toxic dipeptide repeat proteins. This reduction correspondingly mitigated the pathological manifestations characteristic of c9ALS/FTD. Recently, RiboTACs have shown promise as a novel method to convert small molecules with no inherent RNA activity into bioactive degraders. By screening and validating a series of natural product‐like small molecules that specifically bind libraries of RNA motifs, Disney et al. [17] have conjugated these compounds to RNase L recruiters, generating ribonuclease‐targeting chimeras that can selectively degrade RNA targets, including disease‐associated pre‐miR‐155, JUN mRNA, and MYC mRNA, thereby modulating downstream biological functions. These results suggest the efficacy and generalizability of RiboTACs strategy in interrogating RNA‐associated pathways by degrading target RNAs. Further studies may be necessary to expand the chemotypes that can recruit different ribonuclease for different cell lines and to enhance the overall pharmaceutical properties of these heterobifunctional compounds. Furthermore, traditional RIBOTAC strategies face challenges in developing highly specific and selective RNA‐targeting therapeutics, particularly due to the lack of efficient targeting of disease‐specific RNAs. By designing a novel inducible RiboTACs (iRiboTAC), which coupled an RNA G‐quadruplex (G4) binder with a caged ribonuclease recruiter, Zhang et al. [263] enabled on‐demand activation of ribonuclease recruiter through bio‐orthogonal reactions or tumor‐specific triggers. Experimental results demonstrated that iRiboTAC effectively degraded G4 RNA both in vitro and in vivo, specifically inducing cancer cell death while exhibiting strong tumor‐targeting effects and growth inhibition. Further research into the versatility of iRiboTAC strategy might reveal its effectiveness across a broader spectrum of RNA varieties and medical conditions, potentially enhancing its value as a therapeutic approach.

### 6.3 Proximity‐Induced NA Degrader

PINAD is a novel method that uses an imidazole payload, an essential RNA degrading moiety present on many natural ribonucleases, to induce the degradation of specific NAs. It consists of an RNA binding moiety and a linker terminated with an imidazole structure (Figure 5D) [241]. Due to the proton transfer and catalytic capabilities of imidazole, it can initiate degradation reactions in the vicinity of RNA. Therefore, the primary function of imidazole within the PINAD framework is to act as the degradation group. Unlike RiboTACs, where both the RNA‐binding and RNase‐recruiting components could contribute to specificity, PINAD's selectivity relies solely on its RNA‐binding moiety. Therefore, RiboTACs are likely to achieve greater overall selectivity than PINAD. The potential advantage of PINAD is its generally low molecular weight that might facilitate membrane transportation and cellular absorption. Researchers have employed this methodology to engineer degradation agents targeting two distinct RNA structures within the SARS‐CoV‐2 genome: G‐quadruplexes and the frameshifting pseudoknot [264, 265]. Following a comprehensive screening process, several compounds exhibited significant RNA degradation efficacy. Further studies confirmed that these molecules possess the capacity to bind specifically and avidly to target RNA sequences, subsequently inducing their degradation, thus suggesting potential therapeutic applications of PINAD in vivo [241]. The PINAD strategy presents a complementary approach for targeting and degrading diverse pathogenic RNA species to RiboTACs and small‐molecule degraders, potentially expanding the repertoire of druggable targets and addressable pathologies.

The development of small‐molecule RNA degraders represents a transformative approach to modulating RNA function, particularly for disease‐associated targets where functional binding sites remain elusive or are difficult to reach. While these technologies demonstrate promising specificity and efficacy in degrading pathogenic RNAs, challenges such as off‐target effects, optimization of molecular selectivity, and biocompatibility remain to be addressed. Future advancements will require refining degrader design, expanding the repertoire of recruitable nucleases, and improving delivery systems to enhance therapeutic potential. As these innovations progress, RNA‐targeted degradation holds significant promise for treating RNA‐driven diseases, offering a versatile toolkit to explore previously undruggable transcripts and their roles in pathology.

## 7 Targeting the Interface: RPIs

RPIs are critical to numerous cellular processes, including gene expression regulation, RNA splicing, translation, and stability. Understanding the molecular basis of these interactions, which hinge on the specific binding between RNA molecules and RNA‐binding domains (RBDs) within proteins, is essential for elucidating their roles in cellular activities and disease phenotypes. The prediction of RPIs has become increasingly pivotal in bioinformatics and computational biology, where a variety of computational methods have been developed to address the challenges posed by the structural diversity and complexity of RNAs and proteins. Different types of RBDs, such as RNA recognition motifs (RRMs), zinc finger (ZF) domains, and K Homology (KH) domains, are crucial in determining the specificity and strength of RNA binding, thus regulating a wide range of cellular processes. Furthermore, experimental methods for detecting and screening small molecules that modulate RPIs, such as cell‐based screening assays and in vitro fluorescence‐based assays, are key to developing targeted therapies. By integrating computational predictions with experimental screening, researchers can better understand the regulatory mechanisms of RPIs and their implications in disease, leading to the development of novel therapeutic strategies targeting RPIs.

### 7.1 Molecular Basis of RPIs

The molecular basis of RPI involves specific binding between RNA molecules and proteins, often facilitated by RBDs present in proteins [266]. These interactions are crucial for the formation of ribonucleoprotein (RNP) complexes, which play key roles in the regulation of cellular activities and the determination of disease phenotypes. RPIs are central to the control of mRNA metabolism, which includes splicing, export, localization, translation, and degradation. For instance, splicing factors like SR proteins and hnRNPs interact with pre‐mRNAs to modulate splicing outcomes, influencing the diversity of protein isoforms produced by alternative splicing [267]. Similarly, interactions between RBPs and mRNAs can regulate mRNA stability and translation efficiency, thereby controlling the levels of specific proteins within the cell. In the context of translation, RPIs are critical for the initiation process, where proteins such as eukaryotic initiation factors (eIFs) bind to mRNAs to promote ribosome assembly and start codon recognition [268]. Furthermore, the interactions between miRNAs and Argonaute proteins form the core of the RISC, which can bind to target mRNAs and suppress their translation or promote their degradation [269]. Aberrations in RPIs are often associated with various diseases, particularly cancers, neurodegenerative disorders, and viral infections. In diseases like ALS and FTD, mutations in genes encoding RBPs such as TDP‐43 and FUS result in the mislocalization and aggregation of these proteins, disrupting their normal RNA‐binding functions and leading to neuronal cell death [270]. Moreover, many viruses, including HIV and SARS‐CoV‐2, hijack the host's RPI machinery to replicate and evade the immune response [271]. Targeting these virus–host RPIs offers a potential therapeutic strategy for combating viral infections [272]. Advances in structural biology, high‐throughput sequencing, and computational modeling have provided deeper insights into the specificity and dynamics of these interactions, paving the way for innovative drug discovery targeting RPIs in various pathological contexts.

### 7.2 Different Types of RBDs

**FIGURE 6:**
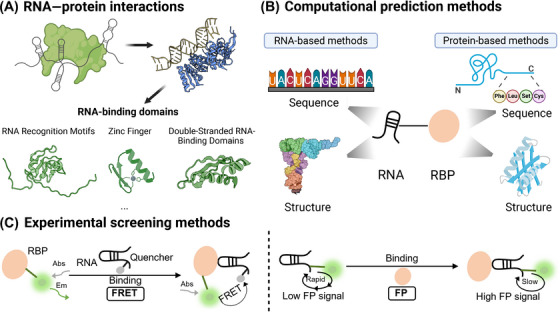
Elucidating, predicting, and targeting RPIs. (A) Molecular basis of RPIs and multiple RNA‐binding domains. (B) Computational prediction methods can be divided into RNA‐based and protein‐based methods. Both sequence and structure features are utilized in the prediction of RPIs. (C) Experimental screening methods including fluorescence resonance energy transfer (FRET) and fluorescence polarization (FP) are employed to detect small molecules that modulate RPIs. Created with BioRender.com.

The molecular basis of RPIs is largely dictated by specific RBDs within proteins. These RBDs are crucial for recognizing and binding RNA molecules with high specificity and affinity, enabling proteins to participate in a variety of cellular processes, including RNA processing, splicing, stability, and translation regulation. Here, we reviewed some of the key RBDs including RRMs, ZF domains, KH domains, double‐stranded RBDs (dsRBDs), Piwi/Argonaute/Zwille (PAZ) domains, and others (Figure 6A), highlighting their structural characteristics and biological functions [273].

The RRM is one of the most common and versatile RBDs, present in approximately 2% of all human proteins. RRMs typically consist of about 90 amino acids forming a β‐sheet structure flanked by two α‐helices. This domain can recognize specific RNA sequences or secondary structures through interactions with the RNA's phosphate backbone and nucleobases. RRMs play pivotal roles in a range of RNA‐related processes, such as RNA splicing, polyadenylation, mRNA stabilization, and translation regulation. The flexibility of RRMs allows them to adapt to various RNA targets, making them integral components of spliceosomal proteins, polyadenylation factors, and other RBPs involved in posttranscriptional gene regulation. ZF domains are small, structural motifs stabilized by the coordination of one or more zinc ions. These domains were initially identified in DNA‐binding proteins but are also crucial in RNA binding. ZFs can recognize specific RNA sequences or structures, playing roles in transcriptional regulation, RNA metabolism, and protein synthesis. The Cys–Cys–His–His ZF, for example, is common in transcription factors that regulate gene expression by binding to both DNA and RNA. Additionally, some ZF proteins, such as ZFP36 (Tristetraprolin), are involved in RNA turnover by binding to AU‐rich elements in the 3’‐UTRs of mRNAs, targeting them for degradation. The KH domain is another widespread RBD, characterized by a conserved sequence motif of around 70 amino acids. KH domains are known for binding single‐stranded RNA, often in a sequence‐specific manner. Structurally, the KH domain adopts a βααβ fold, which forms a cleft where RNA binds. Proteins containing KH domains are involved in critical cellular processes, such as RNA splicing, translation regulation, and mRNA localization. For instance, the fragile X mental retardation protein, which is associated with Fragile X syndrome, contains KH domains that are essential for binding to specific mRNAs and regulating their translation, a function crucial for normal neuronal development. dsRBDs are specialized for binding double‐stranded RNA (dsRNA) structures, which are often found in various RNA species, including precursor miRNAs (pre‐miRNAs) and viral RNAs. The dsRBD typically recognizes the A‐form helical structure of dsRNA through its conserved α‐β‐β‐β‐α fold. Proteins with dsRBDs are involved in RNA interference (RNAi), where they play roles in the processing of pre‐miRNAs by Dicer, and in the function of RISCs. The PAZ domain is a specialized RBD found in Argonaute proteins, which are central to the RISC in RNAi pathways. The PAZ domain binds to the 3' end of small RNAs, such as miRNAs and small interfering RNAs, anchoring them within the RISC. This interaction is crucial for guiding Argonaute proteins to target mRNAs, facilitating their cleavage or translational repression, thereby regulating gene expression. RBDs are integral to the function of RBPs, enabling them to recognize and interact with specific RNA molecules, thereby regulating a wide array of cellular processes. The diversity of RBDs, such as RRMs, ZFs, KH domains, dsRBDs, and PAZ domains, reflects the complexity and versatility of RPIs. These domains not only determine the specificity and strength of RNA binding but also contribute to the dynamic regulation of gene expression at multiple levels, from RNA processing to translation [274]. Understanding the molecular basis of these interactions is essential for elucidating the mechanisms of RNA regulation and for developing therapeutic strategies targeting RPIs in various diseases.

### 7.3 Predicting RPIs

Predicting RPIs has long been a significant focus in bioinformatics and computational biology. Experimental methods like RNA immunoprecipitation and cross‐linking immunoprecipitation followed by sequencing have enabled the accumulation of large datasets of RPIs [275, 276]. However, experimental validation is still time‐consuming and costly, making computational approaches essential for predicting RPIs on a larger scale. Many challenges still exist in this field. First, RNAs and proteins exhibit a wide range of structures, making it difficult to predict interactions based solely on sequence data [277]. Second, although high‐throughput techniques have provided a wealth of data, it often includes false positives or incomplete interaction networks [278]. Third, the interactions are not just binary, they involve multiple factors such as secondary structure, tertiary structure, and the cellular environment. To solve these challenges, various computational methods (Figure 6B) have been developed to predict RPIs, each with distinct approaches and strengths:

- *Sequence‐based methods*. These methods rely on the primary sequences of RNA and proteins to predict interactions. They often involved feature extraction step in which key features such as k‐mers, sequence motifs, and physicochemical properties are extracted from RNA and protein sequences and learning step fulfilled by support vector machine, random forests, and deep learning models trained on known interaction datasets to predict new interactions [279].
- *Structure‐based methods*. These approaches consider the three‐dimensional structures of RNA and proteins: Computational docking tools simulate the binding between RNA and protein molecules to predict interactions. MD simulations provide insights into the stability and conformational changes of RNA–protein complexes over time [280].
- *Hybrid approaches*. Combining multiple data types (e.g., sequence, structure, and network information) enhances prediction accuracy. Integrating various data sources into a unified framework, often using deep learning, to capture the full complexity of RPIs [278, 281]. With the advent of deep learning, models are becoming more accurate in capturing the complex patterns involved in RPIs. Combining RPI data with other omics data (e.g., single‐cell transcriptomics [282], proteomics) is helping to build more comprehensive interaction maps.

As models become more complex, there is a growing need for methods that provide interpretable predictions, allowing biologists to understand the underlying mechanisms. Predicting RPIs remains a challenging but rapidly advancing field. As computational methods continue to evolve, they will likely play an increasingly important role in understanding RNA biology and uncovering new therapeutic targets.

### 7.4 Experimental Methods to Detect and Screen for Small Molecules that Modulate RPIs

To detect and screen for small molecules that modulate RPIs, various experimental methods are employed (Figure 6C) [283]. Recent strategies for targeting RPIs include both RNA‐targeting and protein‐targeting approaches [284]. Cell‐based screening assays involve screening small molecules in living cells to identify modulators of RPIs, providing insights into their functional effects within a cellular context.

For example, the yeast three‐hybrid system uses a hybrid RNA and two fusion proteins to form ternary complex which led to the transactivation of the reporter gene [285]. High‐throughput methods such as in vitro fluorescence‐based assays—including FRET, fluorescence polarization (FP), and fluorescence intensity‐based assays (Figure 6C)—and chemiluminescence‐based assays like the catalytic enzyme‐linked click chemistry assay are used to detect and quantify RPIs and to screen for small molecules that modulate these interactions. For example, a robust FRET assay was utilized to identify small‐molecule inhibitors of LIN28–let‐7 miRNA interactions, which have the potential to serve as novel anticancer agents [286]. Besides, inhibitors of eIF4A, such as rocaglates and pateamine A, have been identified as modulators of translation initiation with concomitant anticancer activities using FP assay [287], with a synthetic rocaglate (eFT226) currently undergoing clinical trials for the treatment of advanced solid tumors, including breast and non‐small cell lung cancers. By predicting RPIs and understanding their regulation and control in cellular activities and disease phenotypes, researchers can develop targeted therapies to modulate these interactions. The combination of experimental and computational approaches enhances our ability to identify small molecules that can specifically target RPIs, offering new avenues for therapeutic intervention.

Despite the advancements in understanding RPIs, the field faces several significant challenges. The extensive structural diversity of both RNAs and proteins complicates the prediction of interactions based solely on the sequence data. Additionally, high‐throughput experimental techniques, while generating vast amounts of data, often suffer from issues such as false positives and incomplete interaction networks, which can hinder accurate RPI modeling. Furthermore, RPIs are influenced by multiple factors, including secondary and tertiary structures, as well as the cellular environment, adding layers of complexity to their study. Looking forward, the development of more precise and interpretable computational models is essential to improve prediction accuracy and biological relevance. Integrating advanced structural biology techniques with high‐throughput data will enhance our ability to decipher the specificity and dynamics of RPIs. Moreover, innovative experimental methods are needed to efficiently screen and validate small molecule modulators of RPIs. A deeper mechanistic understanding of RPIs in various disease contexts will also be critical for identifying new therapeutic targets. As computational and experimental tools continue to evolve, the study of RPIs is expected to play an increasingly vital role in advancing RNA biology and the development of novel therapeutic strategies.

## 8 Conclusion and Prospects

The emergence of RNA‐targeting small molecules marks a paradigm shift in modern drug discovery, offering novel therapeutic avenues for diseases previously deemed undruggable. This review has highlighted the recent progress in understanding RNA structures, the identification of bioactive small molecules, and the integration of computational and experimental methodologies to accelerate the discovery of RNA‐targeting small molecules. From ribosomal RNA‐targeting antibiotics to bioactive splicing modulators, such modality has demonstrated its potential to modulate RNA function with high specificity and efficacy. Recent successes on preclinical and clinical studies that built upon decades of fundamental research, have catalyzed a surge in RNA‐targeting drug development programs, particularly for neurodegenerative disorders, oncology, and rare diseases. With multiple candidates now advancing through clinical pipelines, the coming decade promises to deliver transformative RNA‐targeting therapies for traditionally intractable diseases with unmet medical needs.

Structural determination of RNA targets represents a critical step in developing RNA‐targeting small molecules. While the inherent conformational flexibility of RNA molecules presents substantial challenges for structural characterization, considerable progress has been made through complementary experimental and computational approaches. Advances in structural biology techniques, including X‐ray crystallography, cryo‐EM, and NMR spectroscopy, are generating growing repositories of high‐resolution RNA–ligand complex structures. These structures not only enhance our mechanistic understanding of molecular interactions but also provide valuable datasets for machine learning applications. Recently, Degenhardt et al. [288, 289] introduced HORNET, an integrated RNA structure determination method combining atomic force microscopy with unsupervised machine learning and deep neural networks. Exemplified by this pioneering approach, we are anticipated to witness more integrated approaches that will expand RNA structure determination toolkit, enabling more comprehensive structure characterization and supporting rational design of RNA‐targeting ligands.

Another fundamental challenge in developing RNA‐targeting small molecules lies in defining their distinct chemical properties, given RNA's unique chemical nature. To address this, innovative screening methods such as DELs and fragment‐based approaches have been developed to systematically probe diverse chemical spaces for RNA‐binding compounds. Complementing these experimental strategies, computational methods leveraging cheminformatics and molecular similarity algorithms enable effective exploration of RNA‐biased chemical space, facilitating the curation of FcS libraries for more efficient hit identification.

Looking ahead, the field is poised to explore new frontiers, such as RNA degradation technologies like RiboTACs and proximity‐induced degraders, which offer innovative mechanisms to modulate RNA function. Future development of these bifunctional molecules will likely focus on the drug‐likeness properties, including improved cellular delivery efficiency and minimized off‐target toxicity. The expanding capability to target ncRNAs and untranslated mRNA regions (UTRs) has significantly broadened the therapeutic landscape. Notably, RPIs are emerging as particularly promising targets for pharmacological intervention, offering new mechanisms to modulate gene expression. As our understanding of RNA biology deepens and technological advancements continue, the potential to revolutionize treatment paradigms for diseases becomes increasingly attainable.

In conclusion, the field of RNA‐targeted small molecule discovery stands at an exciting juncture. The convergence of advanced biotechnologies in structure determination, screening methodologies, computational design, RNA degradation, and targeting of specific RNA elements and RPIs heralds a new era in drug discovery. The future of this field lies in addressing existing challenges, fostering innovation, and translating scientific discoveries into transformative medicines. With continued investment and collaboration, RNA‐targeting therapies are set to play a pivotal role in the next generation of precision medicine, offering hope for patients with conditions that have long eluded effective treatment.

## Author Contributions

Zhengguo Cai wrote the “Bioactive small molecules targeting RNA” section, “Screening and hit identification for RNA binders” section and NMR, X‐ray crystallography, chemical probing parts in “RNA structure determination” section, and drew related figures as well as the graphical abstract. Hongli Ma wrote the “Computer‐aided ligand design,” “Targeting the interface: RPIs” sections and drew the related figures. Fengcan Ye wrote the Cryo‐EM and computational approaches in the “RNA structure determination” section, the molecular docking part in the “Computer‐aided ligand design” section, and drew related figures. Dingwei Lei wrote the “Small molecules that degrade RNA” section and drew related figures. Zhenfeng Deng assisted in the writing of “Bioactive small molecules targeting RNA” section. Yongge Li and Ruichu Gu assisted in the manuscript conceptualization and proof‐reading. Zhengguo Cai and Han Wen organized and revised the paper. All authors have read and approved the final manuscript.

## Ethics Statement

The authors have nothing to report.

## Conflicts of Interest

Author Zhengguo Cai, Yongge Li and Han Wen are employees in DP Technology, but has no potential relevant financial or nonfinancial interests to disclose. The other authors have no conflicts of interest to declare.

## Data Availability

The authors have nothing to report.
